# The glass-ceiling convective regime and the origin and diversity of coronae on Venus

**DOI:** 10.1073/pnas.2504491122

**Published:** 2025-09-16

**Authors:** Madeleine C. Kerr, Dave R. Stegman, Suzanne E. Smrekar, Andrea C. Adams

**Affiliations:** ^a^Institute of Geophysics and Planetary Physics, Scripps Institution of Oceanography, University of California San Diego, La Jolla, CA 92093; ^b^Jet Propulsion Laboratory, California Institute of Technology, Pasadena, CA 91109

**Keywords:** Venus, mantle convection, coronae, mineral phase transitions, numerical modeling

## Abstract

Venus and Earth are rocky planets of roughly the same size and bulk density, yet their surface volcanic and tectonic features appear substantially different. On Venus, the coexistence of large volcanic highlands—interpreted as the surface expression of long-lived mantle plumes—alongside coronae, smaller features thought to be caused by transient thermal diapirs, remains enigmatic. Using two-dimensional numerical models of mantle convection with sharp and broad mineral phase transitions for pyrolite, we show that both scales of upwellings can be generated in a stagnant lid planet with an interior temperature 250 to 400 K warmer than Earth’s. The smaller plumes originate from a ~600 km deep internal layer that exists as a consequence of the different sequence of mineral phase transitions that occur in warmer mantles less processed and differentiated by partial melting and volcanism. Future models that include melting will provide further tests of our hypothesis.

## Significance

Venus and Earth are considered to be “twin” planets, sharing roughly the same size, bulk density, and distance from the Sun. Despite our lacking substantial data of Venus’s interior, the planets’ surfaces indicate that they diverged substantially in their evolution. The presence of coronae features on Venus—in contrast to their absence on Earth—is a fundamental mystery in planetary science. This study explains a possible process for how convection in Venus’s mantle can produce small-scale upwellings (~60 to 1,000 km) which generate many coronae, large scale upwellings (~2,000 km) which generate the large volcanic rises, and the observed surface dynamic topography. The presence of the mineral phase transitions underpinning these dynamics argues for a mantle temperature over 200 K hotter than Earth’s.

## Venus’s Plume-Thermal Dichotomy

*Coronae* are volcano-tectonic features arguably unique to Venus, characterized by quasi-circular fracture annulae. There are over 700 globally distributed coronae which exhibit a diverse range of size and topographic morphologies (1). Several of the largest coronae (>500 km in diameter) such as Artemis, Atahensik, and Quetzalpetlatl are proposed to form via large-scale lithospheric instability such as subduction or delamination (2–4). The vast majority of coronae, however, are smaller with a mean diameter of approximately 200 km, and are typically attributed to thermal upwellings such as diapirs or small-scale plumes at different stages of rising and impinging upon the lithosphere (5, 6). The wide range of topographic morphologies have also motivated investigations of the role of melt intrusion (7–9) as well as localized circumferential delamination (10, 11) to produce rims, as well as central lithospheric dripping (12).

Venus’s surface has ~10 large, globally distributed volcanic rises (13) with broad topographic swells approximately 2,000 km across, large positive gravity anomalies consistent with dynamic mantle compensation, and volcanic features consistent with significant pressure-release melting above a plume “head” (14, 15). These features are indicative of long-lived mantle upwellings attached to the hot thermal boundary layer (TBL) at the core–mantle boundary (CMB) region by a sustained mantle plume conduit (“tail”) (e.g. Hawaii or Iceland on Earth). Understanding how coronae *and* large volcanic rises can be simultaneously generated by two distinct scales of mantle upwellings is crucial for understanding the tectono-magmatic system that couples Venus’s surface and interior.

The question of whether the hot TBL at the CMB alone can generate a diversity of mantle plumes is linked to a planet’s convective regime and has been explored via analog fluid experiments (16–18) and conceptually applied to Venus (7). The temperature difference and corresponding viscosity contrast across the CMB influences the frequency and spatial pattern of plume generation, the characteristic size and morphology of these plumes, and their excess temperatures (16, 19). In general, the larger the viscosity contrast, the larger the plumes and the more sustained the conduit (20). On Venus, the temperature and viscosity contrasts may be smaller compared to Earth due to the absence of plate tectonics, where accumulated cold subducted slabs can generate a high temperature and viscosity contrast at the core–mantle boundary (20). Instead, “thermals,” or plumes lacking sustained tails were considered to be the dominant style of plume generation in Venus’s mantle (7, 20). These smaller thermals could be “captured” in larger scale mantle flow and driven to the surface to generate coronae (7, 16, 17).

The coexistence of both large mantle plumes and thermal diapirs originating from a single TBL had been modeled in bottom-heated analog-fluid systems undergoing secular heating (17) as well as during a transition from a mobile-lid regime with large-scale plumes to a stagnant-lid regime with only thermals (18). However, even a 30× increase in the mantle viscosity between the along-adiabat surface and core conditions (*SI Appendix*, Fig. S1), expected from the pressure dependence of mantle viscosity, can suppress the generation of small-scale plumes or thermals at the CMB in favor of fewer, larger mantle plumes (21). Alternatively, smaller compositional (22) or thermal (23) instabilities might originate from the boundary of the upper and lower mantles, fed by stalled or trapped CMB plumes (24). However, the details of how CMB plumes on Venus would stall at that interface and spawn smaller plumes had not been modeled. In this paper, we propose that mineral phase transitions, such as those in the warmer phase-space of equilibrium anhydrous pyrolite, can generate two scales of plumes in a single convective system, even with moderate to high viscosity contrasts across the mantle domain.

## Mineral Phase Transitions in Venus’s Mantle

**Fig. 1.**
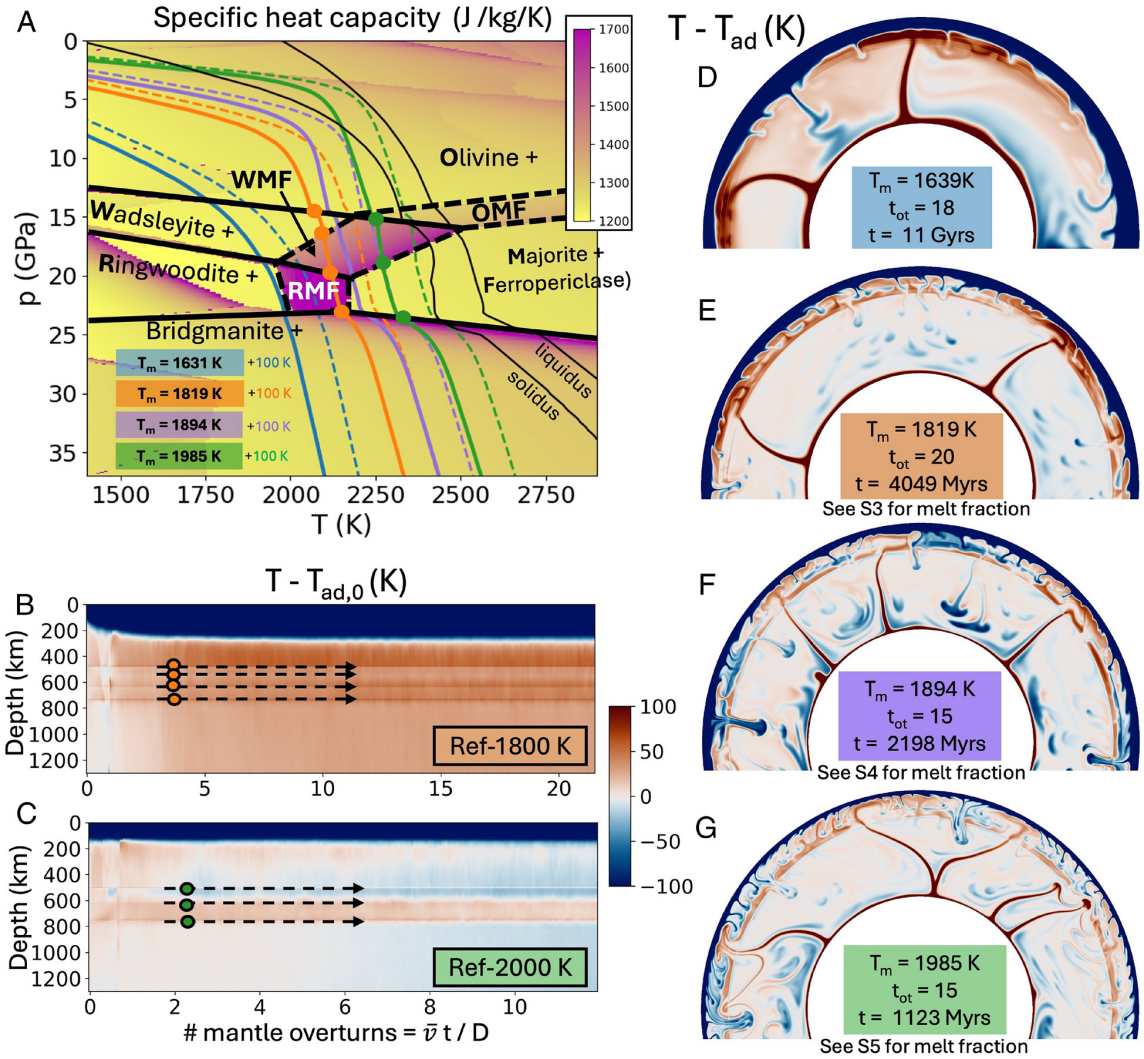
The layering effects of the WMF zone with increasing mantle temperature. (*A*) shows a phase diagram for pyrolite overlaid with average radial temperature profiles (solid-colored lines), liquidus and solidus (from ref. 33) (black solid lines), and the major mineral phases (“+” indicates minor phases) including the wadsleyite to majorite + ferropericlase (WMF) zone. Plots (*B* and *C*) show the time evolution of the average nonadiabatic temperature profiles relative to the initial mantle adiabat Tad,0 for models Ref-1800K (ref. 21) and Ref-2000K. The temperature profiles are taken at the snapshots indicated in (*D*–*G*) and the dashed colored lines indicate temperatures 100 K hotter than the average radial profiles for the four reference models shown. The WMF phase transition zone is easily visualized by its higher specific heat capacity (C_p_) than the surrounding phases, just above the RMF zone (R = ringwoodite) which also has a high C_p_. The orange and green dots correspond to the bounds of the mineral phases crosscutting the profiles (*A*). Plots (*D* and *F*) are the reference 1600K and 1900K models, respectively. Ref-1900K and Ref-2000K show a clear hot-cold boundary around 600 km deep (~18 GPa) indicating a shift into a regime of strong layering of cold drips and mantle avalanches in these warmer mantle temperatures.

The influence of mineral phase transitions on mantle dynamics is a substantially researched topic in numerical geodynamics. Global mantle layering induced by the endothermic reaction of ringwoodite (*ri*) into bridgmanite (*b*g) + ferropericlase (*fp*) at pressures corresponding to 660 km depth on Earth (Fig. 1*A*, blue line) has been shown to occur for Clapeyron slopes <−6.0 MPa/K (25). Clapeyron slopes between −6.0 and −2.0 MPa/K can cause convection to become intermittent with partial layering (25, 26), and in particular, values of −3.5 MPa/K were found to be consistent with the surface topography and geoid of Venus (27). Stagnant or squishy lid planets like Venus (28) have reduced surface heat flows and warmer interiors compared to mobile-lid planets like Earth (28, 29). However, a simplified phase diagram for pyrolite modeled by HeFESTo (30–32) shows that warmer (>1,800 K) temperature profiles (Fig. 1*A*, green line) actually encounter a positive Clapeyron slope when crossing into *bg*, rendering this phase transition unlikely to generate strong layering for dominantly pyrolitic material. Fig. 1 shows that in warmer mantles, olivine and its high-pressure polymorphs, wadsleyite (*wd*) and ringwoodite (*ri*), transform into transitional assemblages of the lower temperature phases (*wd, ri*) and higher temperature phases, majorite (*mj*) and ferropericlase (*fp*) (32). The WMF assemblage (Fig. 1*A*) consists of wadsleyite, majorite, and ferropericlase where the endothermic reaction *wd = mj + fp* occurs.

Both the WMF zone and its high-pressure counterpart, the RMF (*ri = mj + fp*) zone, have a high specific heat capacity (Fig. 1*A*). Additionally, within the WMF zone, an increase in temperature along an isobar corresponds to an increase in the proportion of the denser phases (*mj* and *fp*) at the expense of *wd*. This relationship between temperature and density corresponds to an effective negative coefficient of thermal expansivity (32). This is the same effect exhibited by the *ri = bg+fp* reaction in Earth; however, the zone of reaction for the WMF is broader and the associated Clapeyron slope is more steeply negative (<−10 MPa/K). Although the effects of a broader, yet more steeply negative phase transition (*wd = mj + fp)* on impeding mass flow are greater than those of the *ri = bg + fp* reaction in pyrolite, the WMF zone has been shown to be insufficiently strong to induce global, persistent layering in *mobile-lid* convection simulations, where the effects of the mineral phase transitions on early Earth were explored (34, 35).

Using a numerical model of stagnant lid convection which incorporates the thermodynamics associated with an anhydrous pyrolite mineralogy (34, 36–38 and *SI Appendix*), we investigate whether phase changes in warmer mantles (1,800 to 2,000 K) and the associated global system of ephemeral mantle layering (21) can produce a globally distributed secondary scale of mantle upwellings. We speculate that the phase transition can generate an internal zone of layering along which perturbations from cold lithospheric drips can destabilize the warmer material trapped below and can source the small-scale plumes that produce coronae. To evaluate whether such a layer could reconcile the coexistence of two scales of thermal upwellings, the models include a modest amount of bottom heating (+600 K from the base adiabatic temperature) to generate a few strong plumes from the CMB. These reference models are referred to as Ref-1600K, Ref-1800K, Ref-1900K, and Ref-2000K throughout.

The models are consistent with previous findings (21) that the stagnant lid mode is susceptible to persistent global layering of cold drips in warm pyrolite mantle (Fig. 1 *B* and *C*). In stagnant lid convection, most of the temperature drop across the mantle is locked within the rigid outer shell and unavailable to be recycled into the interior. Therefore, planetary cooling occurs via small drips from underneath the stagnant lid (Fig. 1 *D*–*G*), which have much less negative buoyancy than cold downwellings of mobile lid convection. Fig. 1*B* shows a secondary scale of convection within the top 600 to 740 km of the mantle for the 1800K model. The progression of phase transitions promotes thermal stratification of material warmer than the adiabatic background (Fig. 1*B*), while individual cold drips continue to pass through to cool the deeper interior (Fig. 1*E*). For models with potential temperatures around 1,900 K and 2,000 K, the cold boundary layer grows thinner. The resulting smaller drips (Fig. 1 *F* and *G*) have even further reduced buoyancy and are unable to overcome the barrier to radial flow induced by the phase transition (Fig. 1*C*). Ponded cold material in the WMF zone eventually builds up enough negative buoyancy to produce intermittent flushing events as shown in ref. 21 (Fig. 1 *F* and *G*). These flushing events, called mantle avalanches, have been reported and studied extensively for phase transitions with a negative Clapeyron slope (e.g. refs. 39–41). The drip and avalanche dynamics observed here are robust to varied viscosity variations with depth, lower core temperatures, and a purely internally heated mantle (*SI Appendix*, Fig. S6). The full suite of model results is reported in the Supporting Information.

## Materials and Methods

Compressible thermal convection of Venus’s mantle is modeled in 2D cylindrical annulus geometry using the geodynamic code ASPECT (36, 37), with a core radius of 3,110 km and a planetary radius of 6,052 km. The models have temperature and depth-dependent viscosity which gives rise to a stagnant lid along the surface of the model domain. The governing equations include the entropy formulation of the energy equation (34), the momentum conservation equation, and the mass conservation equation, solved using the projected density approximation (38). The core and surface boundaries are free-slip, and the surface temperature is a constant 740 K for all models, the approximate temperature of Venus’s surface. The suite of numerical experiments includes 15 models in total (Tab. S1) all with a bulk mantle composition of pyrolite. The models are initialized with potential temperatures of 1,600 K, 1,800 K, 1,900 K, and 2,000 K and with constant core temperatures (i.e., bottom boundary conditions) equal to an increase of 600 K (or 300 K, for two models) from the true temperature at the base of the mantle along the initial adiabat. One model has an insulating bottom boundary condition and is heated internally at a constant rate. The models also explore three different degrees of viscosity stratification across the mantle domain: a 30×, 100×, and 1,000× increase between the reference viscosity at the core and the reference viscosity at the surface. Mineral phase transitions within the mantle are incorporated using a precomputed look-up table generated by the software HeFESTo to determine the thermodynamic and physical properties of pyrolite throughout the mantle (30–32). The specific table used here is that which is used in ref. 34, 35 to model early Earth, and then subsequently used in ref. 21 to model Venus. Additional information about the materials and methods can be found in the Supporting Information.

## Discussion and Limitations of the Modeling Approach

Without samples of Venus’ mantle, the major constraints on its bulk composition are the planet’s bulk density (within 10 percent of Earth’s) and its surface being primarily basaltic. The lack of surface water (e.g., ref. 42) may not reflect the current water content in Venus’s interior. For these initial explorations of mineral phase transitions in Venus, we adopt anhydrous pyrolite (30–32), a theoretical, chemically equilibrated composition of Earth’s upper mantle below mid-ocean ridges. Other choices for assumed bulk composition and/or water content likely affect the presence and/or strength of phase transitions and thus the extent of mantle layering. Water content can shift the depths and pressures of mineral phase transitions, introduce new hydrous phases, and depress the melting temperature of mantle silicates, all of which could have a substantial effect on the results presented here. While the effect of water on the WMF transition specifically has not been explored extensively in the literature, ref. 43 notes that for the ringwoodite to bridgmanite and periclase transition (660 km on Earth), the wetness conditions did not affect the magnitude of the Clapeyron slope, although it did broaden the transition. The models in this study also do not incorporate reaction kinetics, which may affect the ability of the coldest downwellings to layer in the upper mantle due to a slower reaction rate as downwellings descend through the depth extent of the MTZ (e.g., ref. 44). A full discussion of the modeling methods is found in the Supplemental Information.

**Fig. 2.**
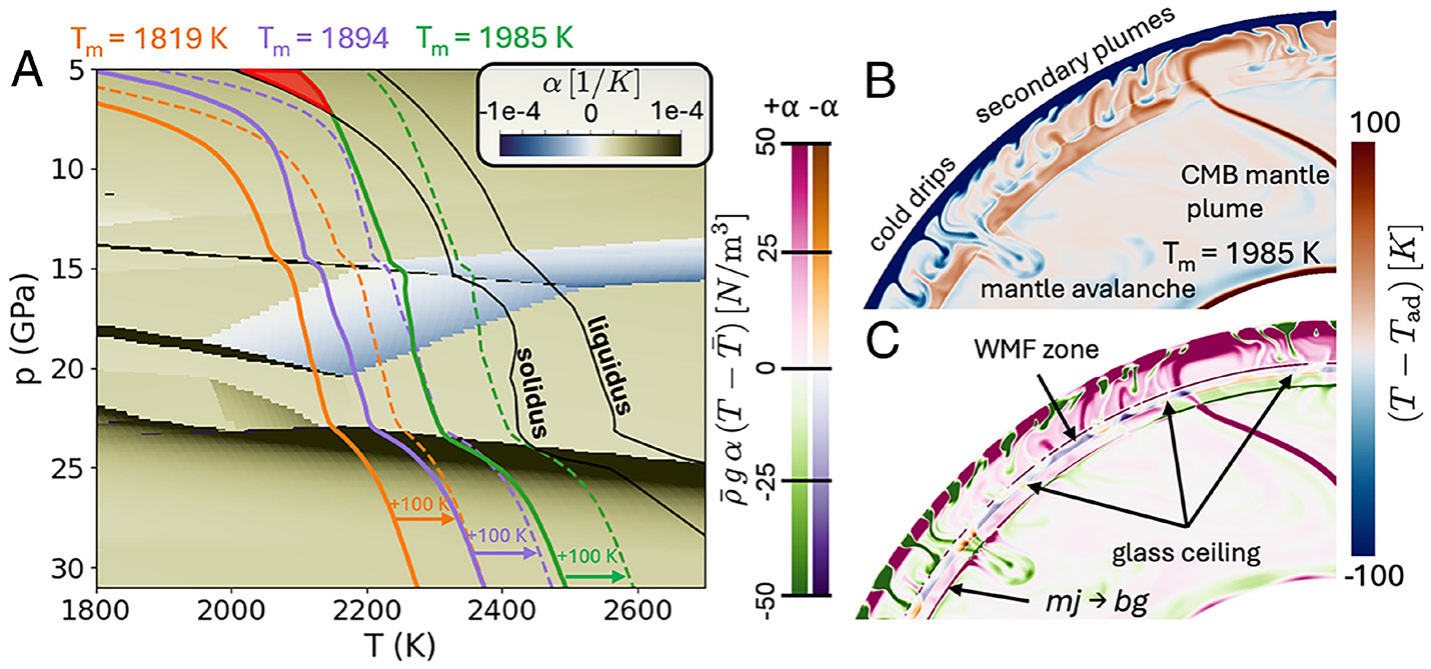
Description of the glass ceiling. (*A*) The thermal expansivity of the multiphase assemblage of pyrolite in p-T space around the WMF zone, overlaid with melting curves (black) from ref. 33. Average radial temperature profiles for Ref-1800K (orange), Ref-1900K (purple), and Ref-2000K (green) are shown with the dotted colored lines noting the temperature +100 K from these mean temperature profiles at 1,819 K, 1,894 K, and 1,985 K, respectively (same as Fig. 1). The snapshot of the Ref-2000K model at 15 overturns shows (*B*) the nonadiabatic temperatures and (*C*) the estimated volumetric buoyancy force for positive (green/pink) and negative (purple/orange) thermal expansivity coefficients. Above 7 GPa in Ref-2000K (*A*, highlighted red), there is global partial melting (*SI Appendix*, Fig. S5) which would generate melt products with different mineral phase equilibria and thermodynamic properties, and which would affect upper mantle dynamics. This diagnostic image shows larger scale convective features (i.e., CMB plumes and mantle avalanches) punch through the glass ceiling while the smaller features (i.e., cold drips) are unable to pass through as readily. As mantle avalanches fall into the lower mantle, trapped warmer material beneath the WMF zone rises as return flow to form secondary plumes.

The numerical techniques used in this study to model the range of pyrolite phase transitions in the relevant p-T space of Venus’s mantle are relatively new (34) and are, for now, limited to the modeling of just a single composition. In general, Venus’s mantle is better described as a mixture of a chemically equilibrated composition (e.g., pyrolite), an enriched partial melt product transported with unknown past-and-present efficiency onto the surface (e.g., basalt), and a depleted remnant left behind in the lithospheric mantle (e.g., harzburgite).The true dynamics of Venus’s mantle likely represent the combined effects of various solid–solid mineral phase transitions and thermodynamic properties for these three (and more) types of distinct compositions which exist in Venus’s heterogeneous mantle. Since the basalt composition in the mantle is often modeled as pyroxene-garnet, the WMF zone is not present for this composition. For harzburgite, the WMF transition zone is pushed to even higher temperatures than in pyrolite due to its relative depletion in aluminum and calcium (see ref. 45, Fig. 2 and ref. 32). Our assumption of a pyrolite source is appropriate if a large portion of Venus’s mantle has not been fully melted, differentiated, and then recycled back into the mantle. The assumption is also suitable if we consider the lithosphere of Venus to be immobile enough that compositional stratification in the lithosphere dominantly occurs where it is stiffer and less coupled to the convecting mantle. Consequently, the uppermost stratified lithosphere may not be incorporated into the drips emerging from the base of the lithosphere.

The evolving tectonic regime of a planet over time influences the compositional components of its mantle. A mobile-lid tectonic regime like on Earth is characterized by frequent subduction and mantle mixing, where it may be more appropriate to model the lower and upper mantles as mechanical mixtures of basalt and harzburgite end-member compositions, as several recent models of Venus have done (e.g., refs. 28, 46, 47). In contrast, ref. 48 calculates that the average Potassium-Uranium (K/U) ratios for Venus’s basaltic crust, along with the total atmospheric argon content, suggest only about 25% of Venus’s total argon has been outgassed from the interior (compared to about 50% on Earth). Is this lower percentage due to an overall reduced amount of melting on Venus, or is it perhaps due to melting concentrated earlier in Venus’s history before most radiogenic argon was formed (48)? In summary, it remains unclear if it is appropriate to model the present-day mantle of Venus as fully processed into a mechanical mixture of partial melt products (i.e., a “marble cake” model of basalt and harzburgite) or as having a substantial mantle reservoir of primordial material dominating the mantle composition of Venus and possibly storing the remaining planetary argon. Our model choices are more in line with the latter assumption and explore convection dynamics within this scope.

If convection on Venus were driven by the sinking of large, cold slabs representing the entire thickness of the lithosphere and consisting of basalt and harzburgite stratified layers, Venus’s mantle would perhaps be more well-mixed than we assume in our convection models. Subduction seems to occur on Venus (2, 3), but not to the extent that it exists on Earth as a part of a plate tectonic system. On Earth, stratified basalt and harzburgite layers compose the upper ~40 km of the oceanic lithospheric mantle (e.g., ref. 49, Fig. 1). If Venus’s lithosphere is similarly stratified, and if the cold thermal boundary layer below Venus’s stiffer lid is below the depth of the harzburgite-pyrolite boundary, then the composition of small mantle downwellings or drips may still be dominantly pyrolitic (49).These drips could then be affected by the negative thermal expansivity in the WMF zone between the 1,700 K adiabat and the solidus (32), and we speculate they would layer in the MTZ. Additionally, there is much left to be discovered about the effect of partial melt, water content, convective dynamics, and mineral grain size on the rates of chemical *re-equilibration* of a basalt-harzburgite mechanical mixture into an equilibrated composition as well as how these processes might work differently under Earth versus Venus conditions (50).

Last, the effect of phase transitions on convective dynamics investigated in this study may change for models that incorporate intrusive volcanism. Plutonism weakens the lithosphere and may generate a “squishy lid” (28) which possibly increases the thickness of the mobile boundary layer at the base of the lithosphere, thus increasing the size of cold drips and thinning the lithosphere. Sublithospheric drips in a plutonic squishy lid regime may entrain a greater percentage of a basalt-harzburgite mechanical mixture into the cold downwellings and induce more melt under the thinner lithosphere. Future studies that track melting in these models would advance our understanding of Venus’s dynamics such as the strength and thickness of Venus’ lithosphere, the size and composition of mantle downwellings, the dynamical effects of multiple compositions passing through the MTZ, the rate of crustal growth, and the total surface heat flow. However, in the simple yet instructive case of 2D stagnant lid convection, incorporating the solid-state phase transitions for equilibrium pyrolite, we encounter parallels between features in our models and observations of Venus’s surface.

### Venus’s Glass Ceiling and Secondary Upwelling Plumes.

The effective negative thermal expansivity of the WMF zone (Fig. 2*A* blue region) plays a larger role in generating mantle layering than other phase transitions with a negative Clapeyron slope in pyrolite. This is because of its greater magnitude and its broader region of reversal from the normal sense of buoyancy in which colder material effectively expands and warmer material effectively contracts (21, 34, 35). In 1800K models (21), the adiabat crosses into the WMF zone where the magnitude of the effective negative thermal expansivity is the smallest. Downwelling material roughly 100 K colder than the adiabatic temperature profile passes through just a thin section of the WMF zone, which mitigates the reversed-buoyancy effect on the downwelling drips. CMB-plumes that enter the WMF zone are strong enough to “punch” through (Fig. 1*E*) and contribute to warming the upper mantle relative to the adiabatic profile which we define by the mean lower mantle entropy.

However, in models with potential temperatures between 1,850 K and the solidus (around 2,100 K) in the transition zone, material within ±100 K of the mean profile is entirely contained within the WMF zone (Fig. 2*A*), with the effective negative expansivity increasing toward the WMF–MF boundary at higher pressures. This produces interesting dynamics: Cold drips sinking from the stagnant lid (Fig. 2*B*) enter the WMF zone and rapidly gain positive buoyancy (Fig. 2*C*), while hot, positively buoyant material rising across the WMF–MF transition becomes negatively buoyant relative to surrounding colder material. The lower edge of the WMF zone at ~600 km depth can be conceptualized as a “glass ceiling,” providing a barrier to flow for both cold and hot material encountering it (Fig. 2*C*). Only the largest CMB-plumes are strong enough to punch through the glass ceiling and form large volcanic rises on Venus’ surface. Most other CMB-plumes are deflected or stall. The trailing conduits in these plumes deliver warm fluid that becomes trapped underneath the glass ceiling (Figs. 1*F* and 2*B*) and flows laterally along its base. This trapped layer is 20 to 50 K warmer than the adiabat but remains subsolidus, relative to anhydrous pyrolite melting curves (33), within the MTZ and the upper mantle.

**Fig. 3.**
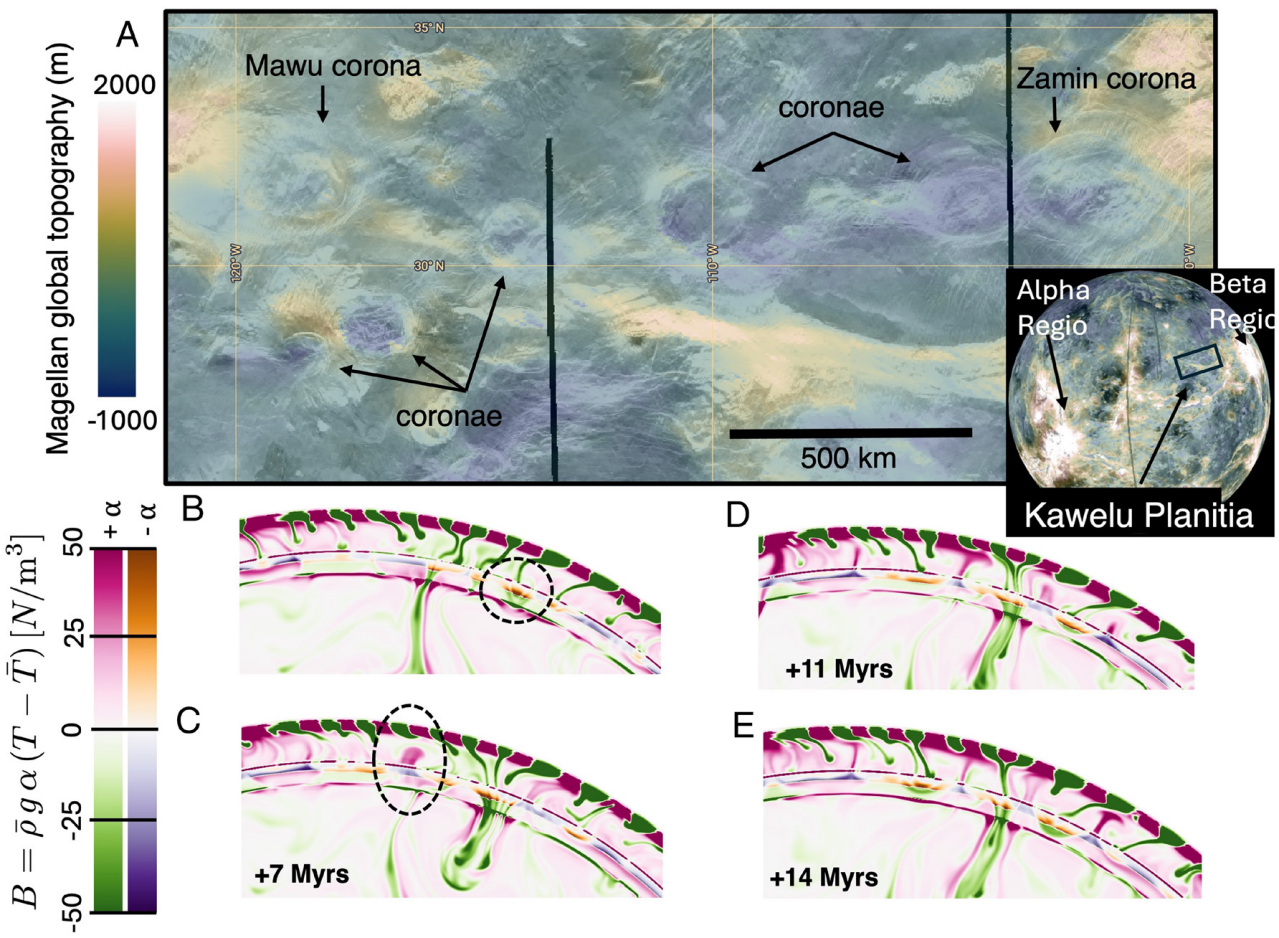
Example of possible coronae formation mechanisms. (*A*) A close-cropped region of Kawelu Planitia on Venus showing a cluster of coronae features (image from Venus Quickmaps). (*B*–*E*) A time evolution of a cold downwelling crossing the glass ceiling and inducing several return-flow secondary upwellings to pass up through the glass-ceiling in response. Because the glass ceiling is not a thermal boundary layer in the classical sense, hot material is first advected as return flow across the WMF region and then can convect upward at ~500 km depth once it regains its sense of “normal” buoyancy. This nonclassical boundary layer behavior allows the secondary upwellings to be diverse in size, even if they are adjacent to one another. The overlapping formation of these features in our 1,900 K and 2,000 K (shown here) models lead us to speculate the secondary upwellings and cold drips may be a formation mechanism for globally distributed coronae of various diameters and morphology.

This layer of warm fluid trapped between 600 to 740 km depth (Figs. 1*C* and 2*B*) provides a global source of smaller-scale thermal instabilities. Flushing events of mantle avalanches form when a critical volume of cold sublithospheric drips accumulate. Associated small, hot plumes are mobilized by the avalanche-driven return flow. These plumes have a wide range of sizes since they do not necessarily obey classical boundary layer theory. The evolution of this process can be seen in Fig. 3 *B*–*E* as a cold drip (circled in Fig. 3*B*) from the base of the stagnant lid critically perturbs the piled-up material in the WMF zone, generating an avalanche which, in turn, initiates four warm upwellings. Many studies that explore the topographic, gravitational, and volcanic features of coronae through geodynamic modeling initiate their regional models with a small hot circular thermal diapir (ranging from 100 km to 260 km in diameter) just below the lithosphere and 100 to 300 K warmer than the surrounding upper mantle (9, 11). Our models provide a simple explanation for the source region of the smaller-scales plumes that are usually imposed as initial conditions for widely cited studies of regional coronae formation, local volcanism, and evolution (9, 11). We conjecture that these secondary plumes—along with their associated uplift, melting, localized delamination, and eventual subsidence—could create the wide range of observed corona morphologies (10) due to phase transitions with thermodynamic properties similar to those of the WMF zone.

### Two Scales of Mantle Downwellings.

Coronae on Venus have highly variable topographic morphology, and therefore the dynamics of their formation are likely equally diverse. In fact, roughly half of all coronae are characterized by central depressions (Fig. 3*A*) and may be the surface expression of smaller mantle downwellings (10, 12). We speculate that coronae characterized by central depressions can be explained as resulting from sublithospheric drips that continuously detach from the cold boundary layer (12). The surface of Venus may record more complex interactions between small-scale drips and ascending secondary plumes as conceptualized in Fig. 3.

Venus’s surface also shows features that indicate a larger scale (1,500 to 2,000 km diameter) of downwelling in the form of circular depressions with associated negative gravity anomalies, such as Atalanta and Lavinia Planitia (e.g., ref. 51). Unlike most plains on Venus, they have almost no coronae or large volcanoes and are dominated by compressional tectonic features (52). The origins of similar scale plateaus of highly deformed terrain (tesserae) are also unknown, though various models have attributed their formation to the surface expression of large-scale mantle upwellings (53) ideally over thin, weak lithosphere (e.g., ref. 54) or downwellings (55). We speculate that the lateral entrainment of cold drips in the upper mantle into the conduit of a mantle avalanche (see ref. 21, Fig. 3*E*) may be the source of compressional features in Atalanta and Lavinia Planitia (56) and the sinking cold mass of the mantle avalanche may generate the observed broad, low geoid anomaly and lower topography.

### Dynamic Topography and Topography Comparisons.

**Fig. 4.**
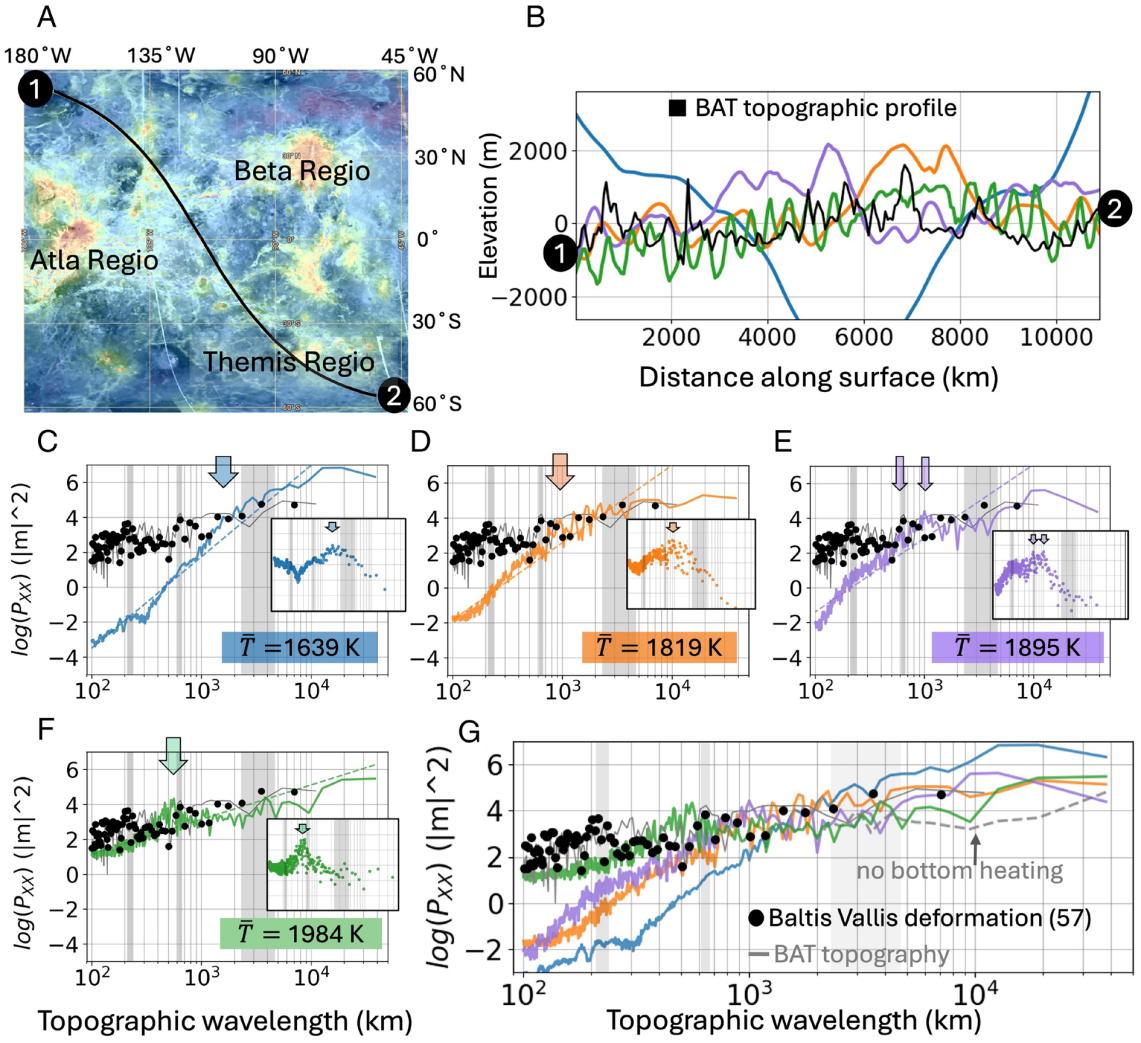
The effect of upper mantle convection on dynamic topography. (*A*) shows a 15,000 km long geodesic profile of Venus’s topography across the volcanically active BAT region (image from Venus QuickMap). The profile is compared to the modeled dynamic topography in the spatial (*B*) and spatial frequency (*C*–*G*) domains. The spatial frequency of the topography in the BAT region, the dynamic topography of the reference models, and the observed characteristic wavelengths of deformation along the Baltis Vallis canali (57) are compared in subplots (*C*–*G*). The light gray bars indicate the range of those characteristic deformation wavelengths on Venus from ref. 57 and the black scattered dots show the processed data from ref. 57 as well. The averaged power of the dynamic topography signals (*C*–*G*) over the course of ~1 mantle overturn (50 timesteps) for the 1,895 K and 1,984 K mantles in a state of steady secular cooling, share some features with the BAT topography and the Baltis Vallis deformation for spatial wavelengths between 400 and 1,000 km. The colored dashed lines represent the power-law fit to the models’ dynamic topography power spectra and the smaller inset subplots show the deviation of the model PSD from the fitted power law. The arrows indicate the wavelengths of the largest positive deviations from that fit. These plots highlight the overlap between the dynamic topography of the models and the Baltis Vallis surface deformation in the last 150 to 500 Mya, particularly the local maxima at 640 km investigated but unexplained by ref. 57.

Our model appears to replicate the intriguing dynamic topography observed on Venus’ plains. The postemplacement deformation of a class of extremely long, narrow lava channels, called canali, manifests topographic change. Baltis Vallis Canali, the longest such example, provides a cumulative record of surface deformation over its 7,000 km length (57). Beyond 950 km (*l*=40), it has been shown the dynamic component dominates the topography (58); however, at shorter wavelengths, the dynamic topography may still have an effect and even control crustal growth (via regional melt) or deformation at spherical harmonic degrees *l* > 40. Fig. 4 *C*–*G* (gray bars) shows the most significant wavelengths of deformation extracted from the Baltis Vallis topographic power spectrum, which may reflect relevant tectonic or mantle processes occurring within the past ~100 to 500 Mya. The shortest length scale peaks (210 to 240 km) were attributed to deformation associated with compressional ridge belts by Conrad and Nimmo (57). The peak at the longest wavelength (3,500 ± 1,200 km) is attributed to the depth of the mantle, although it may approach the limit of the method’s resolution due to the canali’s finite length. They also reported a peak at 640 ± 29 km.

We analyze our model dynamic surface topography in the spatial (Fig. 4*B*) and spatial-frequency (Fig. 4 *C*–*G*) domains alongside a topographic profile of an active region of Venus. The Beta-Atla-T hemis (BAT) region (Fig. 4*A*) is dense in coronae and lacking crustal plateaus. At wavelengths between 400 and 10,000 km, a range generally characterized as being at least partially driven or fully dominated by the mantle dynamic signal due to underlying convection, the 1,895 K and 1,984 K mantles with ~100× viscosity increase across the mantle compare favorably to that of the BAT topography profile (Fig. 4*B*) and the Baltis Vallis dynamic topography (black dots in Fig. 4 *C*–*G*). The 1,639 K and 1,819 K mantles lack a distinct topographic signal centered near 600 km, the length-scale associated with the depth of the glass ceiling.

The initially 2,000 K model, which undergoes secular cooling to 1,984 K, shows a large broad peak in dynamic topographic wavelength between 400 to 1,000 km and centered between 500 and 600 km (green arrow in Fig. 4*F*). We observe a local maximum at ~650 km and ~1,000 km in the 1,900 K model (purple arrows in Fig. 4*E*) along with good agreement between this model, the BAT topography power spectra, and the Baltis Vallis deformation wavelengths from 600 up to 7,000 km. We associate the broad, moderate-wavelength peaks in the 1,984 K and 1,895 K mantles with undulations due to cold drips driving the secondary scale of convection in the upper mantle (Fig. 3 *B*–*E*). As previously noted, a peak in Venus’s dynamic topography is observed at 640 km in the region of Baltis Vallis Canali; however, the authors of ref. 57 did not identify an origin for this feature.

We note that all bottom-heated models show a broad signal in the long-wavelength range, which is attributed to the surface deformation caused by the largest CMB plumes. The dynamic topography reliefs in Fig. 4*B* show the short wavelength oscillations and the longer period oscillations as well. The magnitude of topography at wavelengths between 6,000 km and 20,000 km for the 2,000 K purely internally heated model (Fig. 4*G*, gray dashed line) is flatter and 10 to 100× lower in magnitude. Therefore, we suggest mantle heating in Venus has at least some bottom-heating component to generate the large CMB plumes which drive long wavelength topography, in addition to a secondary scale of convection generating the ~640 km signal seen in Baltis Vallis. These results parallel recent work in ref. 46 which compares the dynamic topography of stagnant-lid mantle convection models to that of Baltis Vallis canali. They show that the age and relief of Baltis Vallis imply a mantle viscosity 1 to 2 orders of magnitude less than Earth’s which they attribute to a less degassed, hydrous interior or to a higher temperature mantle (46). The VERITAS mission to Venus in the next decade will have the ability to better constrain the planet’s mantle viscosity to within a single order of magnitude and its core radius to within ±50 km due to more precise measurements of its complex Love number (59).

While both our study and ref. 46 imply a higher temperature and lower viscosity mantle than Earth’s, our models differ in some baseline assumptions. We speculate a less processed and more primitive mantle composition for Venus whereas ref. 46 assumes a completely processed mantle composed of two end-member compositions: basalt and harzburgite. Thus, the phase transitions between *mj, fp,* and *wd* are shifted to different temperatures and pressures and may vary in their relative strength. Additionally, our models use a 2D cylindrical geometry and do not incorporate melting, the associated crustal growth and heat loss from melting, nor the compositional effects of partial melt differentiation. In general, 2D cylindrical geometry generates some notable deviations in behavior and morphologies from convection in a 3D spherical shell (detailed in ref. 21, section 2.5). For example, “plumes” are infinitely long ‘sheets’ in 2D cylindrical geometry. Recent work on the *wd = mj + fp* phase transition for early Earth indicates that its effects on hot plume laying are stronger in 3D (35). Therefore, we speculate that layering of cold drips, mantle avalanches, and secondary plumes from the glass-ceiling may occur for mantle temperatures outside the bounds of our estimated glass-ceiling regime boundary at 1,850 K.

Last, if the broad dynamic topography signal in our models between 400 and 1,000 km indicates the range of wavelengths for small-scale convection in the upper mantle, the glass-ceiling regime may also provide a conceptual explanation for the enigmatic cratering record of Venus. Different models of steady rate regional resurfacing show that volcanic patches with diameters between 760 to 2,400 km (e.g., ref. 60) or 560 to 2,160 km (e.g. ref. 61) can explain the present-day crater distribution. Secondary convection in the upper mantle due to warm mantle temperatures may drive such regional resurfacing events and explain Venus’s young surface age and crater distribution.

### Conclusions.

The interaction of small, cold drips from the lithosphere and large, hot plumes from the CMB with the WMF zone is perhaps the physical mechanism by which a broader size distribution of mantle upwellings and downwellings are generated, leading to a global landscape of morphologically diverse convection-driven surface features on Venus. Additionally, the glass-ceiling convective regime produces a power spectrum of dynamic topography consistent with some present-day observations of Venus’s recent deformation history. A salient prediction of these models, given our chosen assumptions and physics, is a range of mantle potential temperatures, 250 to 400 K hotter than Earth’s mantle today, within which the glass-ceiling effect operates. It remains unclear for how long in Venus’s lifetime a regime such as this would last, given the narrow range of predicted temperatures (1,850 to 2,000 K), although exploring the dynamics in 3D may broaden this range. There is more work needed to uncover the effect of melting, time-dependent radiogenic heating, and varying core heat flow on the stability of Venus’ internal thermal state. Future work incorporating melting (both intrusive and extrusive), incorporating multiple compositions with distinct phase equilibria, and modeling plume dynamics in 3D over a full planetary evolution timescale will advance our understanding of the interior state and dynamics of Venus.

## Supplementary Material

Appendix 01 (PDF)

## Data Availability

Additional software, model inputs, sample outputs, and data have been deposited in Additional software and data for ‘The glass ceiling convective regime and the origin and diversity of coronae on Venus’ (https://doi.org/10.5281/zenodo.15605469) (62). All study data are included in the article and/or *SI Appendix*. Previously published data were used for this work (https://doi.org/10.26464/epp2024062) (21).
