# Supplementary material for: The glass-ceiling convective regime and the origin and diversity of coronae on Venus
Source: Proc Natl Acad Sci U S A. 2025 Sep 16;122(38):e2504491122. doi: 10.1073/pnas.2504491122 (PMC12478145; doi:10.1073/pnas.2504491122)
Supplement: Supplementary file 1 — Appendix 01 (PDF) [file pnas.2504491122.sapp.pdf]

PNAS

Supporting Information for
The glass ceiling convective regime and the origin and diversity of coronae on Venus

Madeleine C. Kerr[1], Dave R. Stegman[1], Suzanne E. Smrekar[2], Andrea C. Adams[1,2]

[1]Institute of Geophysics and Planetary Physics, Scripps Institution of Oceanography, University of California San Diego; La Jolla, CA, USA.

[2]Jet Propulsion Laboratory, California Institute of Technology; Pasadena, CA, USA

Corresponding author email: mkerr@ucsd.edu

This PDF file includes:

Extended Model Set-up and Methods
Model post-processing
Figures S1 to S7
Tables S1 to S3

Extended Model Set-up and Methods

Using version 2.4 of the geodynamic code ASPECT (1-3), thermal convection of Venus's mantle is modeled in 2D cylindrical annulus geometry with an inner radius (i.e. core radius) of 3110 km and a planetary radius of 6052 km. The mesh consists of quadrilateral finite elements (Q_2 elements for velocity, Q_1 elements for pressure) with a resolution of 128 x 1536 cells, equivalent to about 23 km grid spacing at the surface. The mantle is treated as compressible but with bulk viscosity neglected, and the governing equations include the entropy formulation of the energy equation (4), the momentum conservation equation, and the mass conservation equation, solved using the projected density approximation (5). The equations representing these conservation laws are :

(1) $-\nabla \cdot (2\eta\dot{\epsilon}) + \nabla p = \rho_h \, \boldsymbol{g}$

(2) $\frac{\partial \rho_h}{\partial t} + \boldsymbol{u} \cdot \nabla\rho_h + \rho_h \nabla \cdot \boldsymbol{u} = 0$

(3) $\rho_h \, T \left(\frac{\partial S}{\partial t} + u \cdot \nabla S\right) + \rho_h \, C_p \, \frac{\partial T}{\partial t}\Big|_{\text{cond}} = 2\eta\dot{\epsilon}:\dot{\epsilon}$.

We consider only viscous stresses where the viscosity (η), is a temperature- and depth-dependent Newtonian viscosity, **u** is the velocity vector, $\dot{\epsilon}$ is the deviatoric strain rate tensor which is defined as $\dot{\epsilon} = \frac{1}{2}(\nabla\boldsymbol{u} + (\nabla\boldsymbol{u})^T) - \frac{1}{3}\,(\nabla \cdot \boldsymbol{u})\,\boldsymbol{I}$, p is the pressure (Pa), ρ_h is the hydrostatic density (kg/m^3) which depends on temperature and hydrostatic pressure $\rho_h = \rho(p_h, T)$. S is the specific entropy (J/kg/K); T is temperature (K); and C_p is the specific heat capacity at constant pressure. Note that thermal expansivity is not included explicitly in these equations but is incorporated into the density field at various points in pressure and temperature space which is accessed by using the precomputed HeFESTo look-up table (6-8). For additional details on the derivation and implementation of those respective methods in ASPECT, see ref. 4 and ref. 5.

The suite of numerical experiments described in this investigation includes 15 models in total (see Tab. S1), with 6 that are entirely new (2000 K models) and for comparison, 9 previously published in ref. 9 and run for more overturns in their steady state evolution. It is important to note that these models are not meant to be interpreted as thermal evolution models for Venus, which would require more complex heating modes or evolving temperature boundary conditions at the core. Instead, these models investigate the current interior convective regime of Venus by exploring the convective regimes at snapshots of these models with various interior potential temperatures. Additionally, the data analysis performed on all 15 models is novel to this work, based on methodology developed for this study to both extract and quantitatively assess the dynamic topography from model outputs.

Pyrolite look-up table. Mineral phase transitions within the mantle are accounted by using precomputed look-up tables generated by the HeFESTo software (6-8). HeFESTo computes phase equilibria and physical properties by minimizing the Gibbs free energy for mineral assemblages over the thermodynamic regime of Earth's mantle (6-8). The specific table used here is that which is used in ref. 4 and ref. 10 to model early Earth, and then subsequently used in ref. 9 to model Venus. The specific composition is an equilibrium assemblage of pyrolytic composition consisting of 82% harzburgite and 18% MORB as defined in ref. 11. The current technical limitations of the entropy method in ASPECT are such that only a single mantle composition can be used for a given model. Since Venus's composition is virtually unknown, we use a common Earth-like composition for Venus since the bulk densities of the planet are similar. Future work is expected to investigate the sensitivity of bulk composition to the locations of mineral phase transition in pressure-temperature space. The table, available in the Zenodo repository accompanying this paper, extends from 0−140 GPa in pressure and from 600−3600 J/kg/K in entropy with a resolution in pressure of 100 MPa and 10 J/kg/K in entropy. Since the independent variables of the HeFESTo table are pressure and entropy, and we use the entropy formulation of the energy equation, the values of temperature (T), density (ρ), and specific heat

capacity (C_p) are not computed but instead looked up and thermal expansivity (α) does not make an appearance anywhere in the conservation equations.

Boundary conditions. We use a core radius of 3110 km and a fixed core temperature boundary condition that is set in the model as a fixed entropy boundary condition. The value of that core temperature is equivalent to +600 K (or +300 K in model 2000K-300K) from the initial true temperature at the base of the mantle adiabat, as given in Tab. S2. The planetary radius is 6052 km and has a fixed temperature of 740 K (S =1602.822 J/kg/K at p=0 GPa) for all models. A summary of initial and boundary conditions can be found in Fig. S1.

To set the fixed temperature boundaries using the entropy method, the HeFESTo table is interpolated to convert between entropy and temperature at the surface pressure (p = 0 GPa) and the core pressure (p = 119 GPa). The actual core pressure varies in models that have a different initial density profile (due to a different initial potential temperature/initial entropy) by no more than 1 GPa. Using a pressure of 119 GPa to interpolate the look-up table and set the fixed entropy at the inner boundary generates a difference between the desired and actual temperature difference between the initial mantle and fixed core values of no more than 10 K (<2% for the +600 K models, <4% for the +300 K model). The velocity boundary conditions are free slip for both the inner and outer radii.

Initial conditions. The gravitational acceleration for the models is radially inwards and constant at 8.87 m/s^2. To set the initial mantle potential temperature, we determine which temperature we would like to set (1600 K (see ref. 21), 1800 K (see ref. 21), 1900 K (see ref. 21), or 2000 K), and using the S-p look-up table, we interpolate the equivalent entropy at 0 GPa (i.e. surface pressure). The ASPECT parameter file, when using the entropy method, takes in an initial mantle entropy instead of a potential temperature. It constructs an initial adiabatic temperature field by assigning temperatures at each point from the look-up table using the value of entropy and pressure at that point. Initial mantle entropies are given in Tab. 2.

Viscosity profiles. The mantle viscosity in these models is the product of radially and laterally varying components, as formulated in ref. 12. The radial component of viscosity, $\eta_{rad}(r)$, is based on the initial adiabatic mantle temperature profile, $T_{A,0}(r)$, and the initial hydrostatic pressure profile ($p_{h,0}$) using appropriate temperature- and depth-dependent values for an Arrhenius viscosity. The radial term, which is purely depth-dependent since $T_{A,0}(r)$ and $p_{h,0}$ (r) are fixed profiles, is then multiplied by a factor representing the lateral temperature deviation from the initial adiabat, $\eta_{lat}(T_{A,0}(r), T)$ (12).

$$(4)\quad \eta(T,r) = \eta_{\text{rad}}(r)\cdot\eta_{\text{lat}}(r,T) = A \exp\left[\frac{E+p(r)\,V}{nR\,T_{A,0}(r)}\right] \exp\left[\frac{-H_{\text{lat}}(T-T_{A,0})}{nR\,(T\cdot T_{A,0})}\right]$$

The viscosity is implemented in such a way that $\eta_{\text{rad}}(r)$ is treated as a constant reference viscosity profile. Temperature deviations from the initial adiabat at each azimuthal point and at each depth in time generates lateral variations from that reference profile according to $\eta_{\text{lat}}(r,T)$. The value of H_{lat} is a constant for each model: $H_{\text{lat}} = E_{\text{act}} + 23 \times 10^9\, V_{\text{act}}$, where $E_{\text{act}} = 2.4 \times 10^5$J (13); V_{act} is varied to generate variable viscosity increases with depth and is given in Tab. 2; and 23×10^9 (GPa) represents the approximate pressure of the base of the mantle transition zone where we focus our attention in the models. We consider only diffusion creep ($n = 1$) and A is a constant which defines the viscosity to be 1×10^{21} Pa·s at a surface temperature (p = 0 GPa) of 1600 K. The models lack a sharp jump (i.e. a 100 times increase) from a low viscosity zone in the upper mantle to a higher viscosity in the lower mantle, consistent with the gravity and topography spectra of Venus (14). The low viscosity zones we observe in our models (see Fig. S7) are more gradual increases of viscosity with depth, following the reference Arrhenius profile.

To choose a value of V_{act}, the initial adiabatic temperature ($T_{A,0}(r)$) and pressure ($p_{h,0}$) profiles were extracted from a 1 time-step test model. The ratio of $\eta_{\text{rad}}(r = 3110\text{ km})/\eta_{\text{rad}}(r = 6052\text{ km})$ (not including the viscosity variation due to hot and cold boundary layers) was tested for different

values of V_{act} such that the ratio was 30, 100, and 1000, within 3 significant figures, for all 1600 K, 1800 K, and 2000 K models (Fig. S2). The reference viscosity profiles $\eta_{\text{rad}}(r)$ were written as text files to be read in by ASPECT and are available in the Zenodo repository.

Our model suite spans a range of viscosity profile steepnesses since the absolute and relative viscosity contrasts across the mantle are not well-constrained, even in recent literature (14-17). Most literature focuses on whether there is evidence for a sharp viscosity discontinuity in Venus's mantle. Some argue this discontinuity is in the uppermost mantle (17), others that it is at the upper-lower mantle boundary (15), and others argue there is no discontinuity at all (14). Some investigations give absolute viscosity ranges and others give relative viscosity contrasts.

Our models use the initial mantle adiabatic temperature profile to define a reference viscosity profile which exhibits a spike in the MTZ, for mantles hotter than 1800 K, but is otherwise continuous in the upper mantle and the lower mantle. We note that a low viscosity asthenosphere (e.g., ref. 17) is not ruled out completely by our modeling, especially by the warmest models in our suite which show there would be decompression partial melting just below the lithosphere. We do not implicitly assume this reduction in viscosity in our models, but it will be important to consider in future modeling efforts.

Model post-processing

Mean mantle entropy and evolving reference adiabat T_A. Previous work (9,10) using the entropy formulation of the energy equation in ASPECT defines the non-adiabatic temperature as the real temperature field minus the initial adiabatic profile for a given initial potential temperature. For example, the constant entropy that defined the 1900 K adiabat is 2760.36 J/kg/K, and the value of true temperature at some depth is equal to the temperature of a parcel of material initially 1900 K at the surface and brought down without exchanging heat with the material surrounding that theoretical parcel. In the prior work, that initial 1900 K adiabat would be the reference for visualizations of every future temperature field, however as internal heating and secular cooling occurs, the mean mantle entropy changes as well, informing a new mantle adiabat.

We generate this new adiabatic temperature profile by using the azimuthally averaged entropy values at 500 depth points in the mantle, an output file generated by ASPECT called 'depthaverage.txt'. Between 50 GPa and 90 GPa (below the upper mantle phase transition and above the lower mantle thermal boundary layer and cold piles of remaining downwellings), we take the mean of the mid-mantle entropy values. This lower mantle region is more well-mixed than other regions of the model and is subject to less temperature variations. Using this single value, we use the HeFESTo pyrolite material look-up table (with S and P as independent variables) and interpolate the temperature and density along that constant entropy. This generates a new adiabatic temperature profile which we use to visualize the true non-adiabatic temperature. The visualization process of the estimated thermal buoyancy field uses the mean mantle temperature and density profiles and is shown in Fig 2c. and Fig. 3b-e

Estimated thermal buoyancy. In order to get a better sense of the dynamics of material in and around the WMF zone, a field of estimated thermal buoyancy B_{est} was visualized alongside the non-adiabatic temperature. This volumetric force $B_{\text{est}} = \alpha\rho g\Delta T = \alpha\bar{\rho}g(T - \bar{T})$ treats the current mean temperature and density fields as reference profiles. The estimated thermal buoyancy uses the HeFESTo look-up table to define the effective thermal expansivity α (1/K) at each point. To highlight the unique dynamics of a broad region with a negative effective thermal expansivity, the regions where that coefficient is negative are colored with a different scale to highlight how hot material becomes relatively more dense and cold material becomes relatively less dense than the background temperature at that depth. A previous version of the calculation detailed in ref. 9, Section 3.4 uses the adiabatic temperature and density fields instead, and is a poorer estimate of the volumetric buoyancy field due to the choice of reference temperature and density.

Surface and core conductive heat flow. The surface and core conductive heat flow can be computed given ASPECT model outputs. ASPECT generates .pvtu files for the solution fields and physical parameters which vary in space and time. It also generates a .txt file which azimuthally averages these fields and parameters at evenly spaced depths throughout the 2D annulus domain. Note that for the chosen geometry of 2D cylindrical annulus, the local heat flux across surface elements of the mesh represents the heat flux across a 2D area patch of a 3D infinite cylinder (using translational symmetry along the z axis of the full 3D cylinder) instead of a surface patch on a 3D sphere which is appropriate for planetary geometry. Further discussion on the influence of the 2D cylindrical geometry on model dynamics can be found in ref. 9, Section 2.5 and in ref. 18.

Using the temperature field and the Python package PyVista which handles structured and unstructured meshes as NumPy arrays, the temperature values of a contour of fixed depth (10 km and 2932 km) were extracted and read into a new array along with the azimuthal angle of the data point. The data is sorted and interpolated regularly with spacing of 30 km, since data points on the contours are defined at intersections of the contour and ASPECT finite element mesh edges and are irregularly sampled along the contour. Using Fourier's law of conduction and assuming that 10km above the CMB and below the surface-lithosphere boundary, heat flow is dominated by conduction within these thermal boundary layers, the heat flux is computed as $q = -k \nabla T$. The thermal conductivity (k) is a constant 4.7 J/m/K, and ∇T is approximated by ΔT/Δz where ΔT is the difference between the computed contour and fixed boundary condition temperatures. This gives a heat flux value q at evenly spaced points along the annulus boundaries.

Plots of the surface and core heat flow evolutions are in the extended data and tables section. The snapshots of the models in the paper are all taken at statistical steady-state or quasi-steady-state for each model after the initial transient state where the initial plumes merge and downwellings form, between 1-5 mantle overturn times. Mantle overturn numbers are computed by getting the mean mantle velocity between 50 and 90 GPa for the final 20 outputs in the depthaverage.txt output file. The number of overturns N is equal to this final mean velocity $\bar{v}_f$ times the final output time (t_f) divided by the domain width (6052 km - 3110 km = 2942 km).

Models in the final timestep are in various stages of steady state or quasi-steady state secular cooling or secular heating, depending on the initial mantle temperature and the viscosity contrast across the mantle. Evolutions of the mid-mantle potential temperature and mid-mantle velocity are shown in the extended figures. The models are not meant to show an evolution of Venus from the initiation of mantle convection until today; rather, the models are run to explore the dynamical characteristics of a particular mantle temperature/entropy due to the mineral phases present for those conditions in a composition of pyrolite. Therefore, planetary evolution conditions such as core-cooling or decaying internal heating are not necessary to showcase the specific characteristic dynamics associated with a particular temperature profile and viscosity stratification. Future work should investigate the long lived-ness of the layered regimes as the temperature and the composition of the planet's mantle evolves.

PSD estimation of dynamic surface topography. Evenly spaced dynamic topography data for a snapshot of a given model is produced by reading the full, unevenly spaced dynamic topography data from the surface dynamic topography output, generated by ASPECT, as an array. Since the topographic resolution for the Magellan data is resolved between 10 and 25 km on the surface, we conservatively interpolate the uneven topography profile at evenly spaced intervals of 50 km, with a corresponding Nyquist wavelength of 100 km, the same as the minimum frequency analyzed by ref. 57.

The PSD is estimated with a periodogram (magnitude squared of the discrete Fourier transform (DFT) of the signal) using a Hann window of a length equal to the length of the topography data. These operations are done using the Matplotlib Python package PSD function. The global PSD for the dynamic topography signal of a single model over the course of multiple timesteps is

estimated with Welch's methods (i.e. averaging of the periodograms). We average 50 timesteps which record between 0.8 and 1 mantle overturn at a period of statistical steady state, noting the mean mantle temperature over the course of that overturn.

We compare a linearly de-trended Hann periodogram of sections of Venus topography along geodesics paths (great circle arclengths) to these Welch-averaged Hann periodograms of our global model topography signal. We also compare our dynamic topography signals to the dynamic topography signal obtained for the Baltis Vallis region in ref. 19.

Visualizing the partial melt field. While decompression melting and volcanism are not incorporated into these ASPECT models in either a two-phase flow or parameterized sense, the solidus and liquidus of this pyrolite composition can be used to estimate the degree of melt generation in the model. The partial melt at all points in the model domain is calculated from the melting curves provided in ref. 20 (given in [T, p] coordinates) by computing the extent to which a temperature lies between the solidus and liquidus at a given pressure. Snapshots of the partial melt field for the models are shown in the extended figures section. Additionally, regions of partial melt indicate regions where there is likely to be crustal growth on the surface above. The wavelengths of secondary convection correlate to undulations in depth of the lithospheric base and, in hot mantles, the degree of decompression melting at that location. Therefore, wavelengths of dynamic topography in mantles hotter than ~1900 K also indicate wavelengths of possible increased crustal growth, which allow comparisons between model dynamic topography and Venusian topography in short topographic wavelengths <1000 km (as shown in Fig. 4).

Data and Code Availability Statement:

All 15 models described in this paper were run using the geodynamic code ASPECT v2.4.0 which is available to download from the ASPECT GitHub repository. The parameter files used to run these 15 models (including the 9 models published ref. 9) are all available on a Zenodo repository created to accompany this paper (10.5281/zenodo.14846607). All models were run on the National Energy Research Scientific Computing Center (NERSC) Perlmutter machine on 1 node with 128 cores (1 node = 2 AMD EPYC 7763 (Milan) CPUs (64 cores each) = 128 cores). Models were run for computational time between 10-36 hours. Data was stored on the NERSC scratch storage system and downloaded to a personal MacBook computer for analysis. Extended Fig 1 was created using Paraview v5.11.1. All other data analysis was conducted using Python (analysis scripts in Zenodo) v3.12.2 with packages Pyvista v0.43.7, Matplotlib v3.8.4, Numpy v1.26.4, Crameri scientific computing color maps v1.19, and Scipy v1.2.0.

Venus's topography data from the Magellan Global Topography dataset, was acquired using the website Venus QuickMap (https://venus.quickmap.io), a collaboration between NASA GSFC& Applied Coherent Technology Corp. The *.csv file in the Zenodo repository can be uploaded onto the website by finding the "Layers" tab, finding the "+" symbol in the upper right of that tab, clicking "Import Vector Data," and selecting the csv file from the Zenodo you would like to visualize on the map. The Magellan global topography and SAR imagery data is originally published by the USGS Astrogeology Science Center (21).

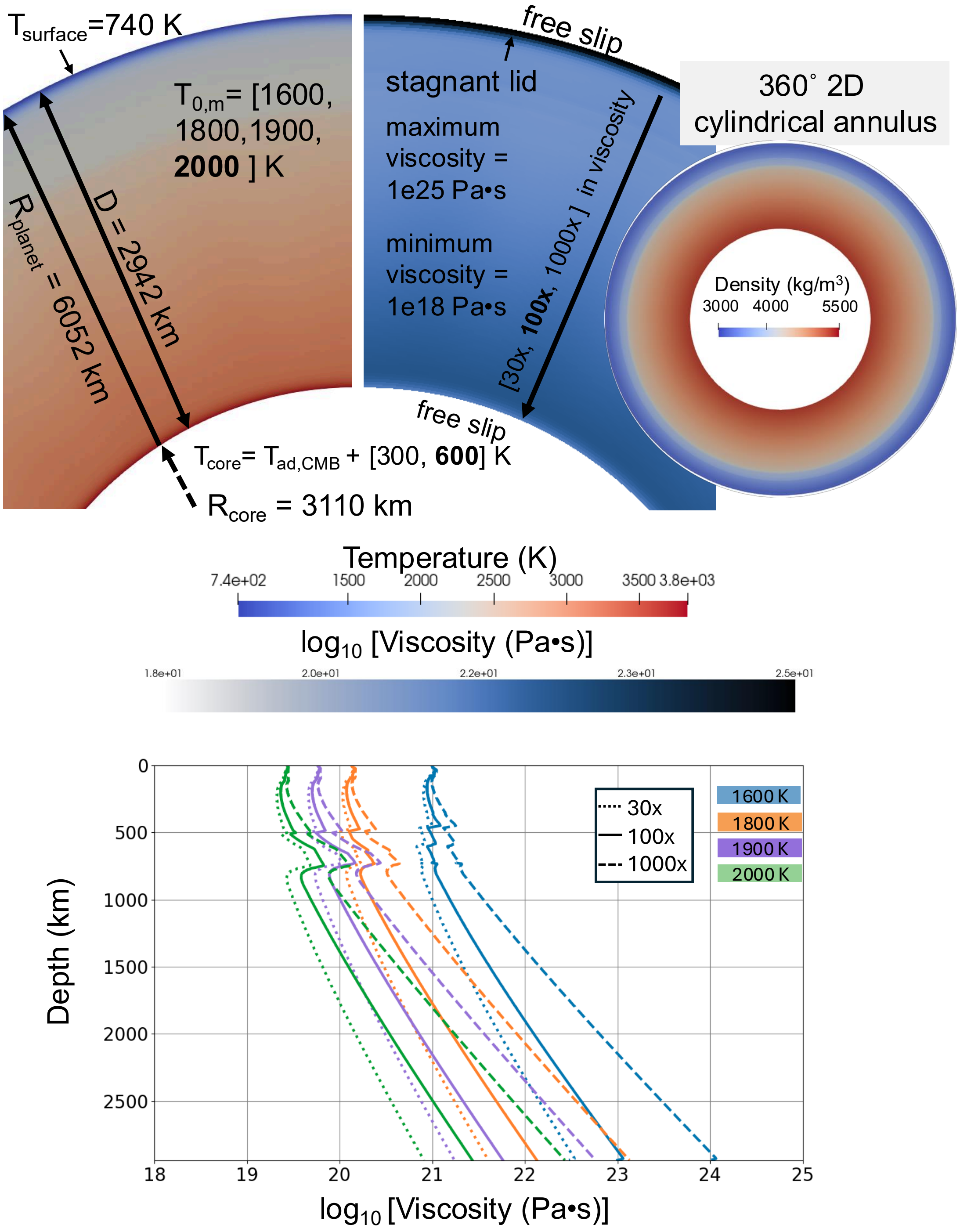

Fig. S1. Summary of initial conditions, boundary conditions, and model geometry. (Top) Temperature, viscosity, and density fields at the first time-step for the 1800 K models with 100x viscosity contrast across the mantle. (Bottom) Reference viscosity profiles for the 1600-2000 K models with varying degrees of stratification.

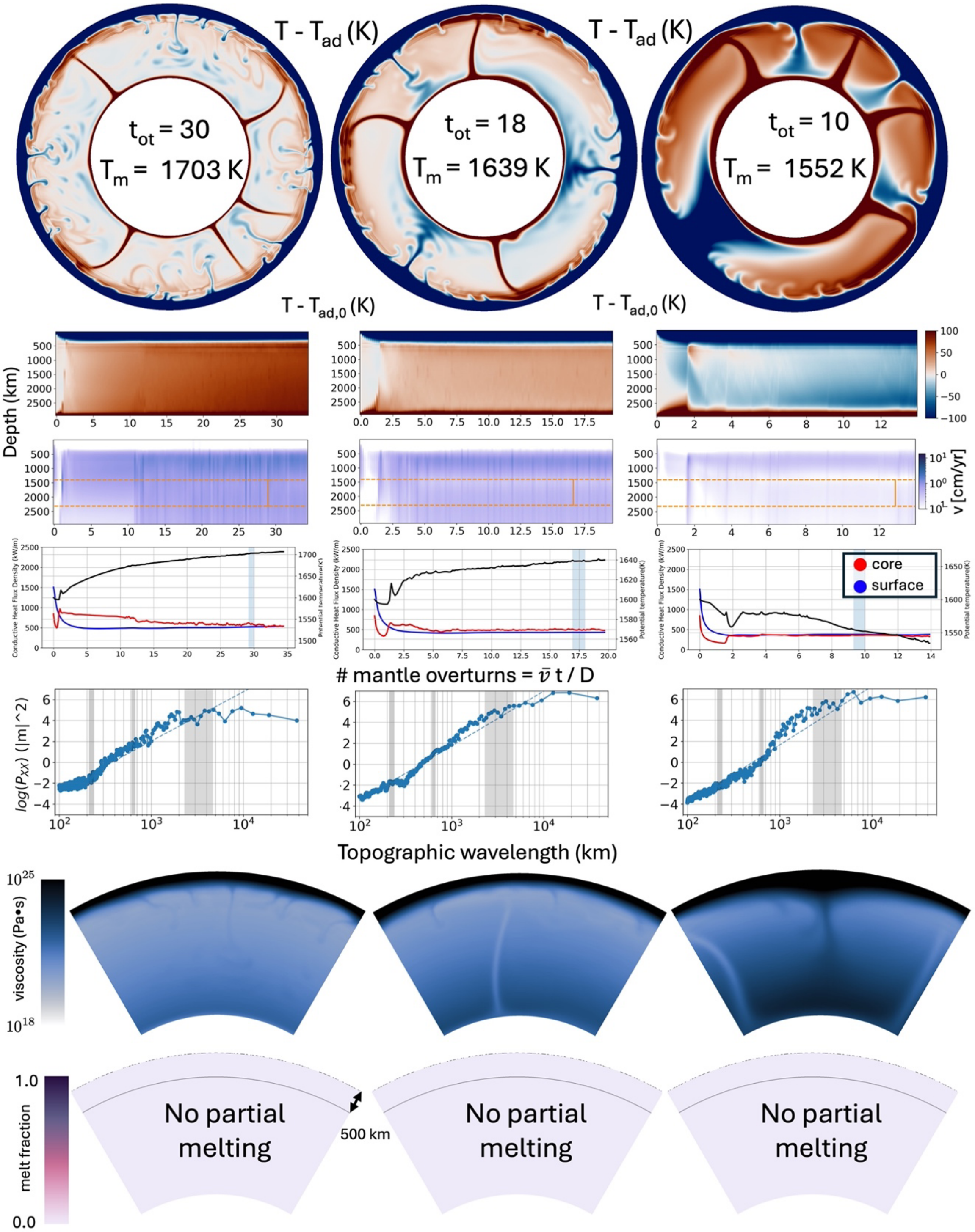

Fig. S2. Model diagnostics for the 1600 K models with 30x (left), 100x (middle), and 1000x (right) viscosity stratifications across the mantle. (From top to bottom) (1) The non-adiabatic temperature field; (2) The laterally averaged temperature deviation from the initial adiabatic profile; (3) the laterally averaged velocity where the average of points in the right-most orange box represents the mean velocity used to compute the overturn time for each model; (4) The core and surface conductive heat flux density and mantle potential temperature evolutions, defined from the mean mantle entropy between 50-90 GPa (orange lines in velocity plot); (5) Power spectra of the

surface dynamic topography averaged between all timesteps shaded in (4), with Baltis Vallis observations (ref. 19) of dynamic topography in gray; (6) wedge subsection of viscosity field; (7) wedge subsection of partial melt field.

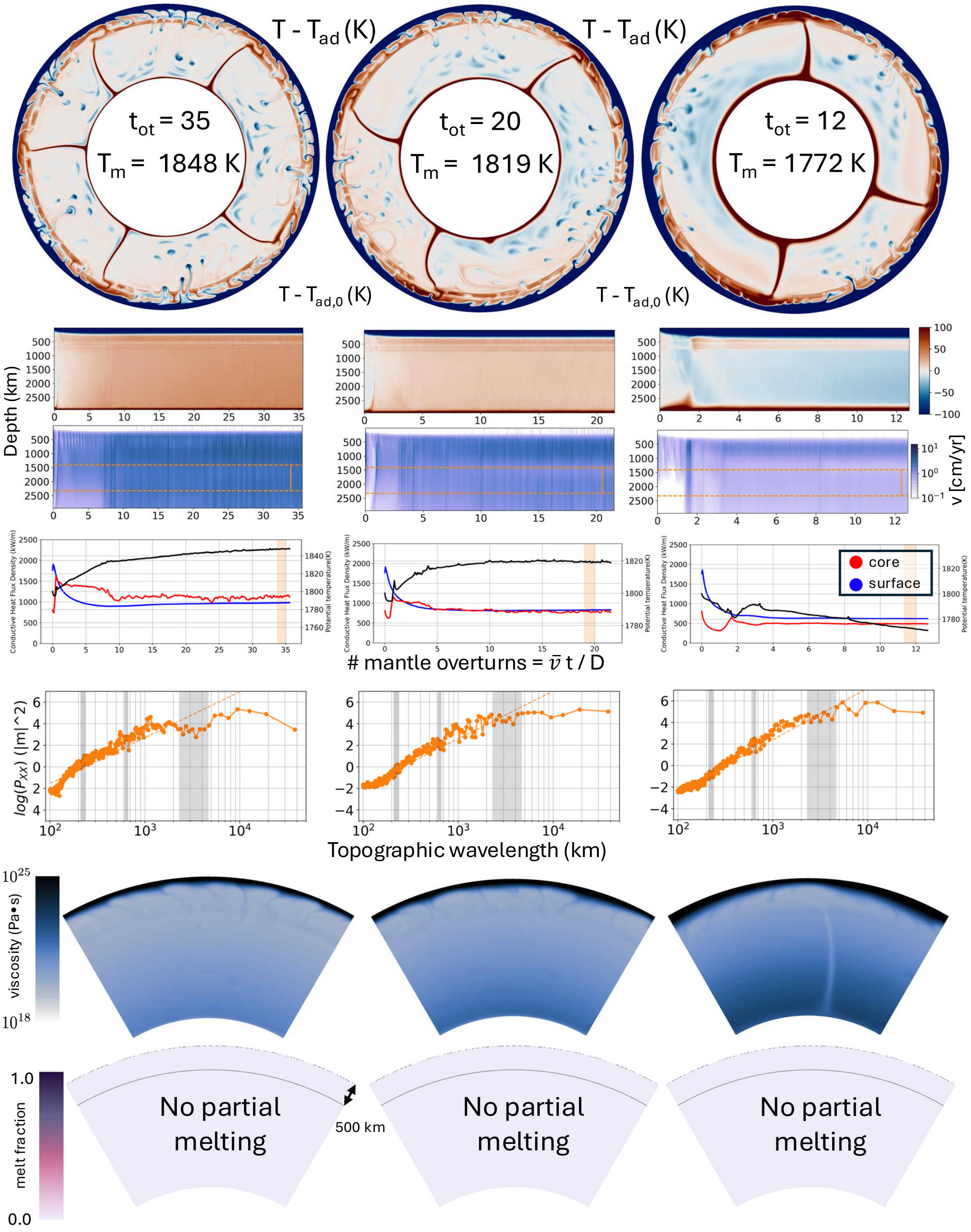

Figure S3. Model diagnostics for the 1800 K models with 30x (left), 100x (middle), and 1000x (right) viscosity stratifications across the mantle. (From top to bottom) (1) The non-adiabatic temperature field; (2) The laterally averaged temperature deviation from the initial adiabatic profile; (3) the laterally averaged velocity where the average of points in the right-most orange box represents the mean velocity used to compute the overturn time for each model; (4) The core

and surface conductive heat flux density and mantle potential temperature evolutions, defined from the mean mantle entropy between 50-90 GPa (orange lines in velocity plot); (5) Power spectra of the surface dynamic topography averaged between all timesteps shaded in (4), with Baltis Vallis observations (ref. 19) of dynamic topography in gray; (6) wedge subsection of viscosity field; (7) wedge subsection of partial melt field.

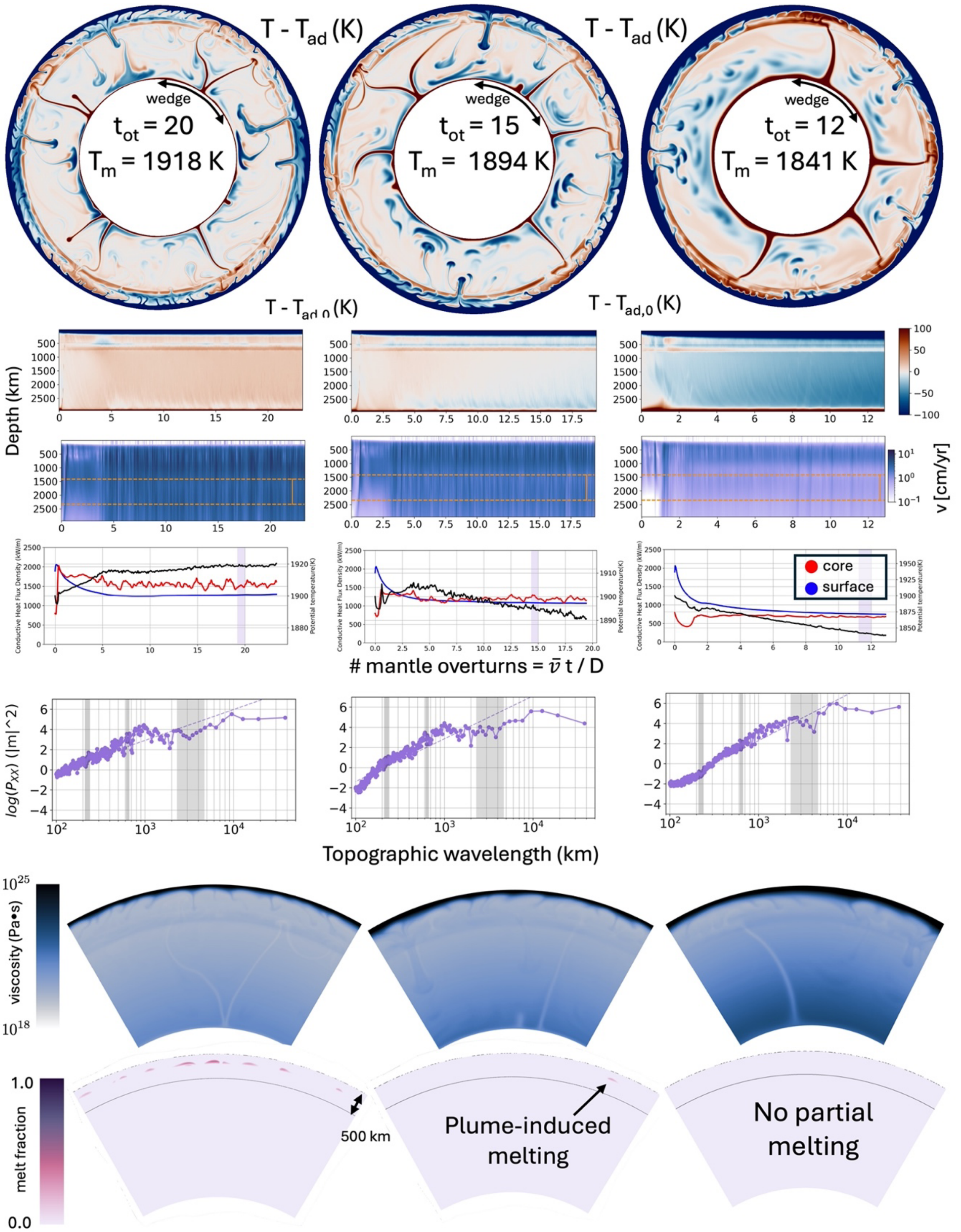

Figure S4. Model diagnostics for the 1900 K models with 30x (left), 100x (middle), and 1000x (right) viscosity stratifications across the mantle. (From top to bottom) (1) The non-adiabatic temperature field; (2) The laterally averaged temperature deviation from the initial adiabatic profile; (3) the laterally averaged velocity where the average of points in the right-most orange box represents the mean velocity used to compute the overturn time for each model; (4) The core and surface conductive heat flux density and mantle potential temperature evolutions, defined from the mean mantle entropy between 50-90 GPa (orange lines in velocity plot); (5) Power

spectra of the surface dynamic topography averaged between all timesteps shaded in (4), with Baltis Vallis observations (ref. 19) of dynamic topography in gray; (6) wedge subsection of viscosity field; (7) wedge subsection of partial melt field. Note the wedges are rotated clockwise to capture the partial melt of upwelling plume heads.

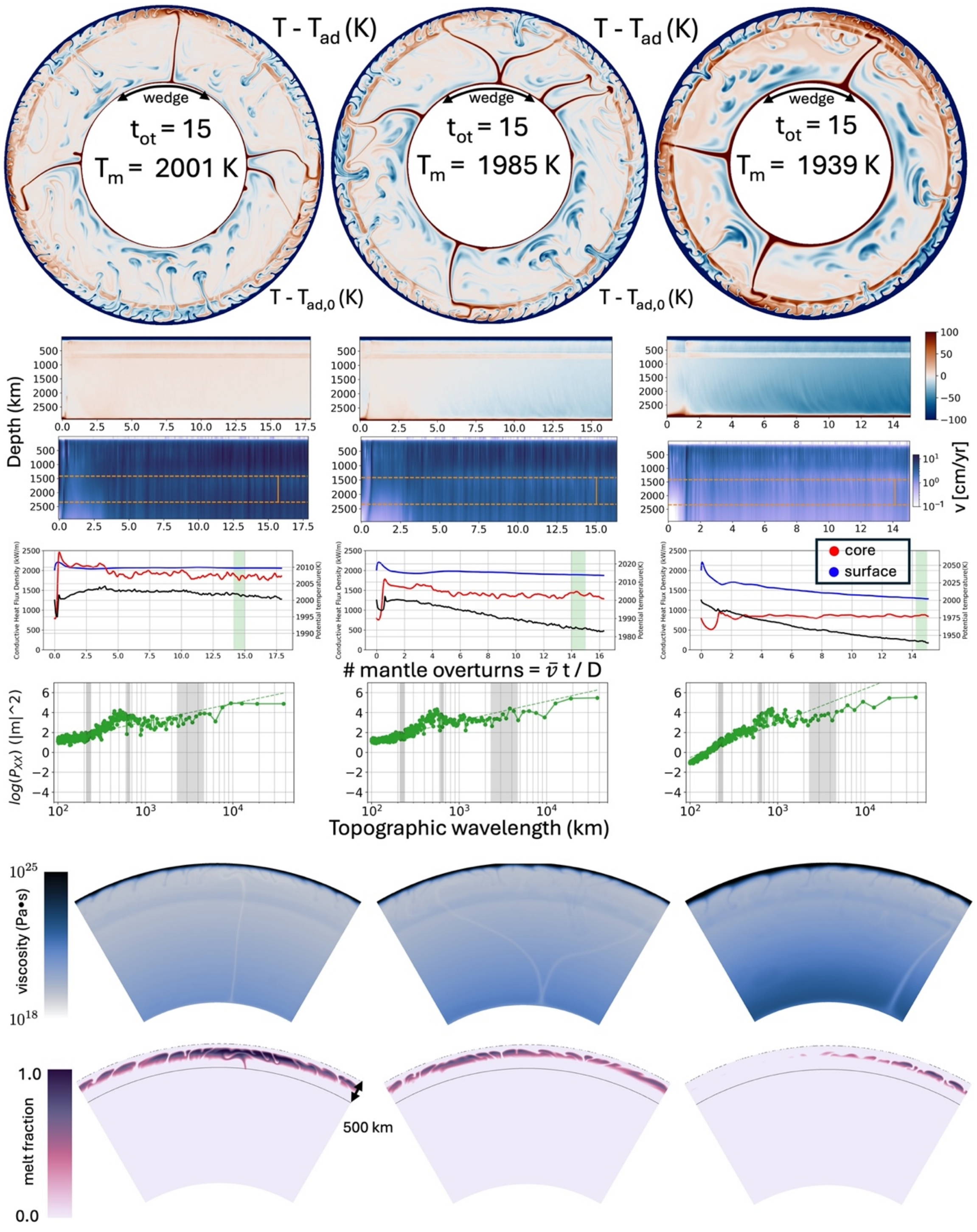

Figure S5. Model diagnostics for the 2000 K models with 30x (left), 100x (middle), and 1000x (right) viscosity stratifications across the mantle. (From top to bottom) (1) The non-adiabatic temperature field; (2) The laterally averaged temperature deviation from the initial adiabatic profile; (3) the laterally averaged velocity where the average of points in the right-most orange box represents the mean velocity used to compute the overturn time for each model; (4) The core and surface conductive heat flux density and mantle potential temperature evolutions, defined from the mean mantle entropy between 50-90 GPa (orange lines in velocity plot); (5) Power

spectra of the surface dynamic topography averaged between all timesteps shaded in (4), with Baltis Vallis observations (ref. 19) of dynamic topography in gray; (6) wedge subsection of viscosity field; (7) wedge subsection of partial melt field.

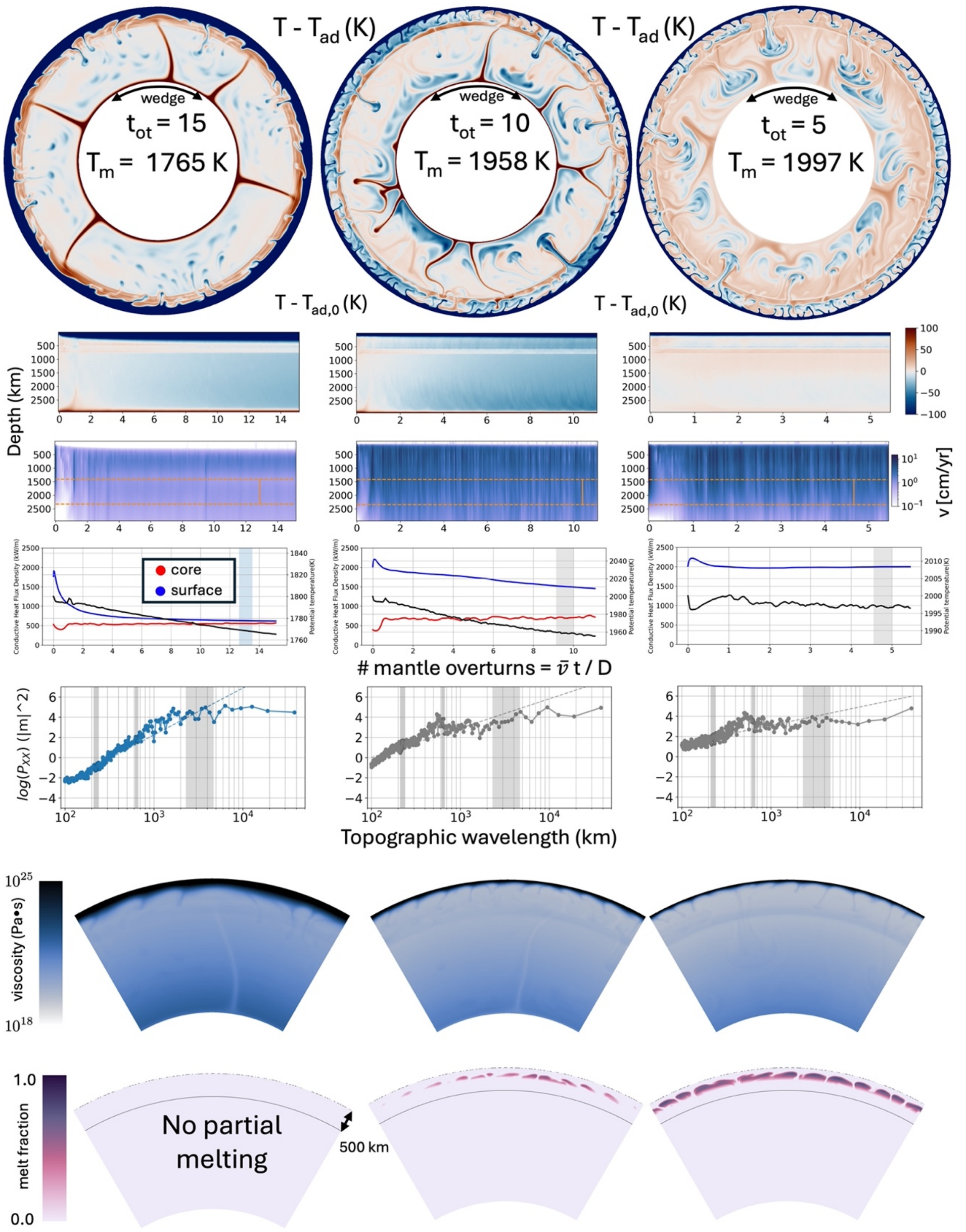

Figure S6. Model diagnostic figure for the 1800 K model with 100x viscosity stratification across the mantle (left) and the 2000 K model with 100x viscosity stratification across the mantle (middle), both with bottom heating where $T_{core} = T_{ad,CMB} + 300$ K. (right) A purely internal heated model starting at 2000 K and with a 100x viscosity stratification across the mantle. (From top to bottom) (1) The non-adiabatic temperature field; (2) The laterally averaged temperature deviation from the initial adiabatic profile; (3) the laterally averaged velocity where the average of points in the right-most orange box represents the mean velocity used to compute the overturn time for

each model; (4) The core and surface conductive heat flux density and mantle potential temperature evolutions, defined from the mean mantle entropy between 50-90 GPa (orange lines in velocity plot); (5) Power spectra of the surface dynamic topography averaged between all timesteps shaded in (4), with Baltis Vallis observations (ref. 19) of dynamic topography in gray; (6) wedge subsection of viscosity field; (7) wedge subsection of partial melt field.

$\Delta T_{CMB,0} = 600\ K$

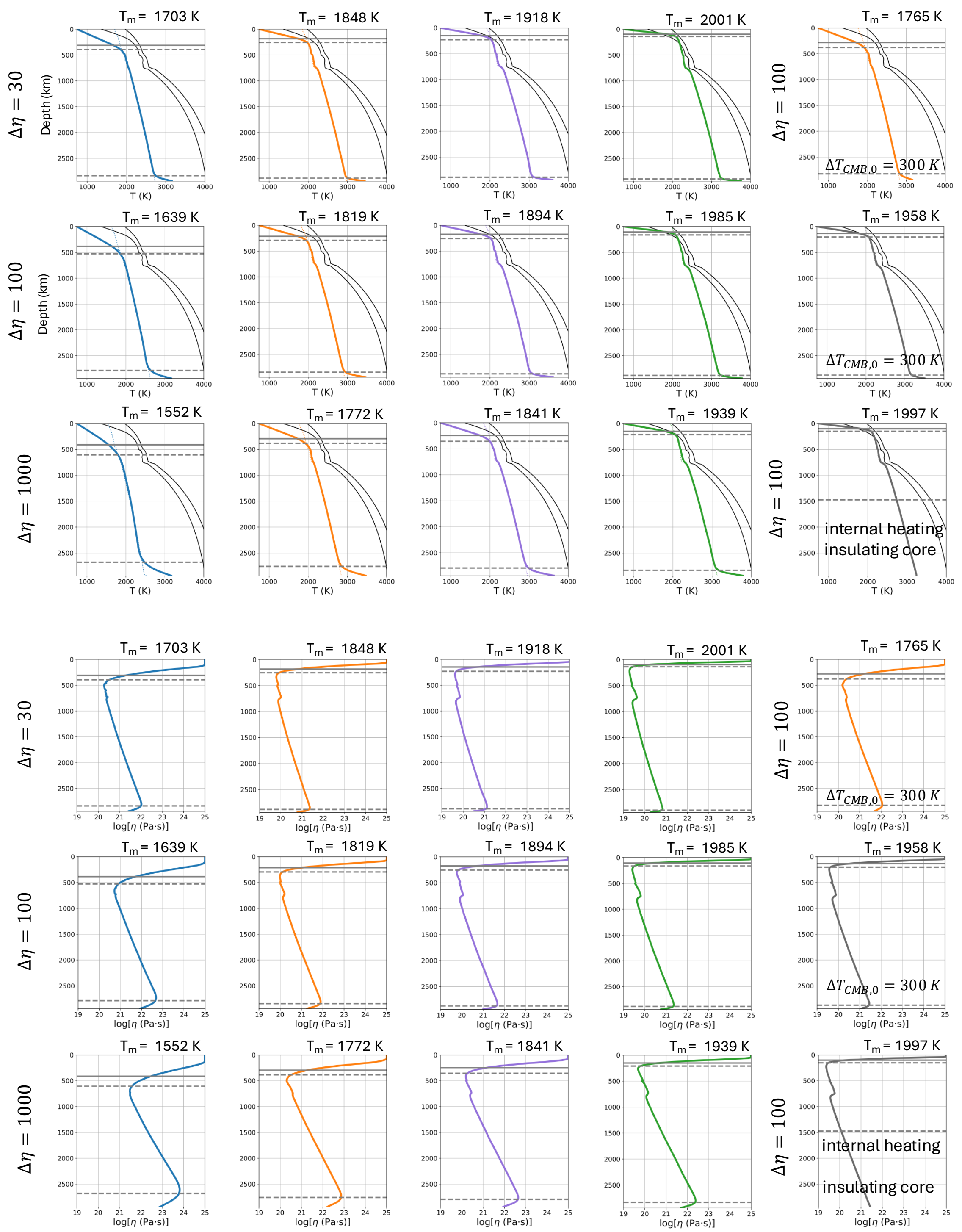

Figure S7. Average temperature (above) and viscosity (below) profiles for all 15 models at the characteristic times denoted in Fig. S2-S6. In the upper 15 plots, the solid black lines are the solidus and liquidus from ref. 20. The solid gray line represents the base of the stagnant lid which is defined as the depth where the mean azimuthally averaged viscosity is 10x greater than the minimum azimuthally averaged viscosity in the domain. The gray dotted lines represent the cold and hot thermal boundary layers computed as a (+/-)10% deviation of the temperature from the adiabatic temperature profile. The adiabatic temperature profile is given by the thin dotted lines.

Table S1. *Physical and thermodynamic parameters and initial/boundary conditions*

Model Parameter	symbol	value [units]
Planetary radius	R_{planet}	6052 [km]
Core radius	R_{core}	3110 [km]
Surface Temperature	T_{surf}	740 [K]
Surface Entropy (@ p=0)	S_{surf}	1602.822 [J/kg/K]
Thermal conductivity	k	4.7 [W/m/K]
Density	ρ	defined in lookup table [kg/m^3]
Thermal expansivity	α	defined in lookup table [1/K]
Specific heat capacity	C_p	defined in lookup table [J/kg/K]
Initial entropy perturbation	--	± 10 [J/K]
Wavelength of entropy perturbation	*l*	23
Mantle potential temperature at time *j*	$T_{j,m}$	1600, 1800, 1900, 2000 [K] (*j=0*)
Mean mantle entropy at time *j*	$S_{j,m}$	Defined in Table 2
Temperature along adiabat at 119 GPa (base mantle pressure varies with $T_{0,m}$)	$T_{ad,CMB}$	Defined from mantle potential temperature and look-up table
Fixed lower boundary condition	T_{core}	$T_{ad,CMB}$ + 600 K
Fixed lower boundary conditions	S_{core}	From T_{core} and look-up table.

Table S2. *Varied initial and boundary conditions*

Model name	$T_{m,0}$	T_{core}	$S_{m,0}$	S_{core}	V_{act}	$T_{ad, CMB}$	$\Delta T_{CMB,0}$
	[K]		[J/kg/K]		[m^3 /mol]	[K]	
1600K-30x	1600	3177.97	2535.079	2802.615	1.92e-6	2576.12	601.85
1600K-100x (Ref)	1600	3177.97	2535.079	2802.615	2.05e-6	2576.12	601.85
1600K-1000x	1600	3177.97	2535.079	2802.615	2.47e-6	2576.12	601.85
1800K-30x	1800	3486.90	2687.72	2920.014	1.92e-6	2890.74	596.16
1800K-100x (Ref)	1800	3486.90	2687.72	2920.014	2.16e-6	2890.74	596.16
1800K-1000x	1800	3486.90	2687.72	2920.014	2.63e-6	2890.74	596.16
1900K-30x	1900	3656.08	2760.36	2980.340	1.98e-6	3063.40	592.68
1900K-100x (Ref)	1900	3656.08	2760.36	2980.340	2.24e-6	3063.40	592.68
1900K-1000x	1900	3656.08	2760.36	2980.340	2.74e-6	3063.40	592.68
2000K-30x	2000	3830.53	2831.62	3039.992	2.05e-6	3241.88	588.65
2000K-100x (Ref)	2000	3830.53	2831.62	3039.992	2.32e-6	3241.88	588.65
2000K-1000x	2000	3830.53	2831.62	3039.992	2.85e-6	3241.88	588.65
1800K-300K	1800	3190.74	2687.72	2801.696	2.16e-6	2890.74	279.52
2000K-300K	2000	3541.88	2831.62	2938.154	2.32e-6	3241.88	289.65
2000K-int	2000	3830.53	2831.62	3039.992	2.32e-6	3241.88	588.65

Table S3. *Model properties at the final timestep of each model.*

Model name	t_f	$\bar{v}_f$	$N_{vt/D}$ ($t_{ot,f}$)	$T_{m,f}$	$S_{m,f}$	$q_{surf,f}$	$q_{core,f}$
	[Myrs]	[cm/yr]	[]	[K]	[J/kg/K]	[mW/m^2] (mean ± std)	
1600K-30x	12600	0.804	34.4	1706.5	2617.9	14.3±2.1	27.6±6.5
1600K-100x	12400	0.465	19.7	1639.8	2566.5	11.4±3.1	24.8±8.4
1600K-1000x	25100	0.163	13.9	1534.3	2481.6	10.1±3.2	17.1±6.3
1800K-30x	4240	2.47	35.6	1848.0	2722.8	25.9±3.6	57.3±13
1800K-100x	4356	1.45	21.5	1819.0	2701.7	21.9±3.3	39.6±8.4
1800K-1000x	7556	0.493	12.7	1771.1	2666.4	16.2±4.1	24.9±5.8
1900K-30x	1912	3.59	23.3	1920.4	2775.0	33.9±4.9	82.7±28
1900K-100x	2843	2.01	19.4	1890.6	2753.7	28.2±4.8	58.8±16
1900K-1000x	4714	0.803	12.9	1838.4	2715.8	19.6±4.4	35.1±9.2
2000K-30x	808.7	6.51	17.9	2000.3	2831.8	54.1±4.9	95.0±27
2000K-100x	1220	3.93	16.3	1983.1	2819.7	49.3±5.6	65.8±16
2000K-1000x	3215	1.38	15.0	1939.1	2788.3	33.8±5.6	43.2±9.7
1800K-300K	5166	0.861	15.1	1765.3	2662.1	16.3±2.2	29.0±5.0
2000K-300K	1561	2.09	11.1	1955.4	2800.0	38.3±3.7	36.8±11
2000K-int	671.8	2.38	5.4	1996.5	2829.1	52.5±4.5	--

SI References

1. T. Heister, J. Dannberg, R. Gassmöller, W. Bangerth, High Accuracy Mantle Convection Simulation through Modern Numerical Methods – II: Realistic Models and Problems." *Geophysical Journal International* **210(2)**, 833–851 (2017). doi:10.1093/gji/ggx195.
2. M. Kronbichler, T. Heister, W. Bangerth, High Accuracy Mantle Convection Simulation through Modern Numerical Methods." Geophysical Journal International **191 (1),** 12–29 (2012). doi:10.1111/j.1365-246x.2012.05609.x
3. A. Glerum, T. Heister, R. Myhill, R. Gassmöller, J. Naliboff, M. Fraters, J. Dannberg, W. Bangerth, ASPECT version 2.4.0. (2022). doi: 10.5281/zenodo.6903424
4. J. Dannberg, R. Gassmöller, R. Li, C. Lithgow-Bertelloni, L. Stixrude, An entropy method for geodynamic modelling of phase transitions: capturing sharp and broad transitions in a multiphase assemblage. *Geophysical Journal International* **231**, 1833-1849 (2022). doi: 10.1093/gji/ggac293
5. R. Gassmöller, J. Dannberg, W. Bangerth, T. Heister, R. Myhill, On formulations of compressible mantle convection. *Geophysical Journal International* **221**, 1264-1280 (2020). doi:10.1093/gji/ggaa078
6. L. Stixrude, C. Lithgow-Bertelloni, Thermodynamics of mantle minerals—I. Physical properties. *Geophysical Journal International* **162**, 610-632 (2005). doi: 10.1111/j.1365-246X.2005.02642.x
7. L. Stixrude, Lars, C. Lithgow-Bertelloni, Thermodynamics of mantle minerals-II. Phase equilibria. *Geophysical Journal International* **184**, 1180-1213 (2011). doi:10.1111/j.1365-246X.2010.04890.x
8. L. Stixrude, C. Lithgow-Bertelloni, Thermal expansivity, heat capacity and bulk modulus of the mantle. *Geophysical Journal International* **228**, 1119-1149 (2022). doi: 10.1093/gji/ggab394
9. M. C. Kerr, D. R. Stegman, Mantle avalanches in a Venus-like stagnant lid planet. *Earth and Planetary Physics* **8**, 686-702 (2024). doi: 10.26464/epp2024062
10. R. Li, J. Dannberg, R. Gassmöller, C. Lithgow-Bertelloni, L. Stixrude, How phase transitions impact changes in mantle convection style throughout Earth's history: From stalled plumes to surface dynamics. *Geochemistry, Geophysics, Geosystems*, **26(2),** e2024GC011600. (2025).
11. W. Xu, C. Lithgow-Bertelloni, L. Stixrude, J. Ritsema, The effect of bulk composition and temperature on mantle seismic structure. *Earth and Planetary Science Letters* **275**, 70-79 (2008).
12. B. Steinberger, A. R. Calderwood, Models of large-scale viscous flow in the Earth's mantle with constraints from mineral physics and surface observations. *Geophysical Journal International* **167**, 1461-1481 (2006). doi: 10.1111/j.1365-246X.2006.03131.x
13. S. I. Karato, P. Wu, Rheology of the upper mantle: A synthesis. *Science* **260(5109)**, 771-778 (1993). https://doi.org/10.1126/science.260.5109.771
14. T. Rolf, B. Steinberger, U. Sruthi, S. C. Werner, Inferences on the mantle viscosity structure and the post-overturn evolutionary state of Venus. *Icarus* **313**, 107-123 (2018). doi: 10.1016/j.icarus.2018.05.014
15. C. Saliby, A. Fienga, A. Briaud, A. Mémin, C. Herrera, Viscosity contrasts in the Venus mantle from tidal deformations. *Planetary and Space Science* **231**, 105677 (2023).
16. Y. Musseau, G. Tobie, C. Dumoulin, C. Gillmann, A. Revol, E. Bolmont, The viscosity of Venus' mantle inferred from its rotational state. *Icarus* **422***,* 116245 (2024). doi: 10.1016/j.icarus.2024.116245
17. J. S. Maia, M. A. Wieczorek, A. C. Plesa, The mantle viscosity structure of Venus. *Geophysical Research Letters***, 50(15),** e2023GL103847 (2023).
18. A. Fleury, A. C. Plesa, C. Hüttig, D. Breuer, Assessing the accuracy of 2-D planetary evolution models against the 3-D sphere. *Geochemistry, Geophysics, Geosystems* **25**, p.e2023GC011114 (2024).
19. J. W. Conrad, F. Nimmo, Constraining characteristic morphological wavelengths for Venus using Baltis Vallis. *Geophysical Research Letters* **50** (2023). doi: 10.1029/2022GL101268

20. L. Stixrude, N. de Koker, N. Sun, M. Mookherjee, B. B. Karki, Thermodynamics of silicate liquids in the deep Earth. *Earth and Planetary Science Letters* **278**, 226-232 (2009). doi: 10.1016/j.epsl.2008.12.006
21. We use the Venus Magellan Global Topography 4641m (link: https://astrogeology.usgs.gov/search/map/Venus/Magellan/RadarProperties/Venus_Magellan_Topography_Global_4641m_v02) and the Venus Magellan SAR FMAP Left Look Global Mosaic 75m, both pusblished by USGS Astrogeology Science Center (https://astrogeology.usgs.gov/search/map/Venus/Magellan/Venus_Magellan_LeftLook_mosaic_global_75m).
